# Species classifier choice is a key consideration when analysing low-complexity food microbiome data

**DOI:** 10.1186/s40168-018-0437-0

**Published:** 2018-03-20

**Authors:** Aaron M. Walsh, Fiona Crispie, Orla O’Sullivan, Laura Finnegan, Marcus J. Claesson, Paul D. Cotter

**Affiliations:** 1 Teagasc Food Research Centre, Moorepark, Fermoy, Co. Cork, Ireland; 2 APC Microbiome Institute, University College Cork, Co. Cork, Ireland; 3 Microbiology Department, University College Cork, Co. Cork, Ireland

**Keywords:** Shotgun metagenomics, Sequencing platform comparison, Low-complexity microbiome

## Abstract

**Background:**

The use of shotgun metagenomics to analyse low-complexity microbial communities in foods has the potential to be of considerable fundamental and applied value. However, there is currently no consensus with respect to choice of species classification tool, platform, or sequencing depth. Here, we benchmarked the performances of three high-throughput short-read sequencing platforms, the Illumina MiSeq, NextSeq 500, and Ion Proton, for shotgun metagenomics of food microbiota. Briefly, we sequenced six kefir DNA samples and a mock community DNA sample, the latter constructed by evenly mixing genomic DNA from 13 food-related bacterial species. A variety of bioinformatic tools were used to analyse the data generated, and the effects of sequencing depth on these analyses were tested by randomly subsampling reads.

**Results:**

Compositional analysis results were consistent between the platforms at divergent sequencing depths. However, we observed pronounced differences in the predictions from species classification tools. Indeed, PERMANOVA indicated that there was no significant differences between the compositional results generated by the different sequencers (*p* = 0.693, *R*^2^ = 0.011), but there was a significant difference between the results predicted by the species classifiers (*p* = 0.01, *R*^2^ = 0.127). The relative abundances predicted by the classifiers, apart from MetaPhlAn2, were apparently biased by reference genome sizes. Additionally, we observed varying false-positive rates among the classifiers. MetaPhlAn2 had the lowest false-positive rate, whereas SLIMM had the greatest false-positive rate. Strain-level analysis results were also similar across platforms. Each platform correctly identified the strains present in the mock community, but accuracy was improved slightly with greater sequencing depth. Notably, PanPhlAn detected the dominant strains in each kefir sample above 500,000 reads per sample. Again, the outputs from functional profiling analysis using SUPER-FOCUS were generally accordant between the platforms at different sequencing depths. Finally, and expectedly, metagenome assembly completeness was significantly lower on the MiSeq than either on the NextSeq (*p* = 0.03) or the Proton (*p* = 0.011), and it improved with increased sequencing depth.

**Conclusions:**

Our results demonstrate a remarkable similarity in the results generated by the three sequencing platforms at different sequencing depths, and, in fact, the choice of bioinformatics methodology had a more evident impact on results than the choice of sequencer did.

**Electronic supplementary material:**

The online version of this article (10.1186/s40168-018-0437-0) contains supplementary material, which is available to authorized users.

## Background

Next generation sequencing has revolutionised microbiological research by enabling high-throughput metagenomic analysis of mixed microbial communities from many different environments [1–3]. Briefly, metagenomics involves the culture-independent analysis of genomic DNA isolated from an entire microbial community, whereas genomics involves the culture-dependent analysis of genomic DNA isolated from a single microbial isolate [4]. Metagenomic sequencing is an umbrella term which encompasses two distinct culture-independent sequencing approaches: amplicon sequencing or shotgun metagenomics. To date, amplicon sequencing, primarily of the 16S rRNA gene, has been the most commonly utilised metagenomic approach [5]. 16S rRNA gene sequencing is used to investigate the bacterial composition of samples [6], but it is typically limited to genus-level identification [7], although higher resolution is sometimes possible [8, 9]. In contrast, shotgun metagenomics enables species-level [10], and potentially strain-level, classification [11–14] of microorganisms. Importantly, shotgun metagenomics can also be applied to determine the genetic content of samples to assess the associated functional potential [15]. Shotgun metagenomics has been relatively underutilised, primarily because it is more expensive than 16S rRNA gene sequencing as it necessitates considerably higher sequencing depths [16]. Indeed, desired sequencing depth is a factor that frequently dictates the choice of sequencing platform for high-throughput sequencing investigations [17].

A variety of sequencing platforms is currently available from several manufacturers, which vary in sequencing chemistry, read length, and/or throughput. Presently, Illumina sequencers are the most commonly used sequencing platforms for microbiological research applications, including shotgun metagenomics [18]. Illumina sequencing chemistry is based on sequencing-by-synthesis, wherein adaptor-ligated DNA fragments on the surface of a flow cell are amplified by bridge PCR to generate clusters which are then sequenced via cyclic rounds of single-base extension with a mixture of fluorescently labelled dNTPs whose incorporation is detected using a high-sensitivity camera [19]. The Illumina range of sequencers includes, in order of throughput, the MiSeq, NextSeq, and HiSeq series. Generally, the NextSeq or the HiSeq are preferred to the MiSeq for shotgun metagenomics, although there are several examples of the MiSeq also being used for this approach [20–22].

The Ion Torrent PGM from Life Technologies is another frequently utilised sequencer in microbiology, particularly for whole genome sequencing analysis of microbial isolates [23], although it is also used for shotgun metagenomics [24]. In contrast, the higher-throughput Ion Proton, also from Life Technologies, is comparatively overlooked for metagenomic sequencing, whereas it is widely used for exome sequencing analysis of higher organisms [25–27]. Ion sequencing chemistry is based on semiconductor sequencing, wherein adaptor-ligated DNA fragments attached to the surface of beads are amplified using emulsion PCR [28]. Subsequently, these beads are placed inside microwells on a semiconductor sequencing chip, where a sequencing-by-synthesis reaction occurs which is similar to the Illumina method, except that base incorporation is determined by the measurement of pH changes caused by the escape of hydrogen ions during DNA extension.

Numerous studies have previously compared the performances of the Illumina MiSeq versus the Ion Torrent PGM to determine the relative accuracy of the sequencers, and now, it has been well established that the error rate of the Illumina platforms, less than 1%, is lower than that of their Ion counterparts, approximately 1.7% [29]. Specifically, Ion reads contain a higher incidence of insertions/deletions [30], and they are susceptible to premature sequence truncation [31]. Long homopolymer tracts are especially problematic for Ion sequencing [32].

Previous investigations have aimed to determine if the choice of sequencing platform significantly influences metagenomic analyses. Recently, Fouhy et al. compared the MiSeq with the PGM for 16S rRNA gene sequencing analysis and reported that compositional results differed depending on the platform used [33]. However, when these platforms were compared with the HiSeq for shotgun metagenomic applications, it was apparent that compositional results were similar across platforms but varied depending on the species classification tools used [34]. Although these studies focused on gut microbial populations, shotgun metagenomics also has enormous potential with respect to the analysis of low-complexity microbial communities, such as those in foods. Indeed, shotgun metagenomics has already vastly improved our knowledge of the microbiology of a number of fermented foods [35] and has numerous potential applications relating to food quality and safety [36]. Furthermore, it has been proposed that metagenomic analysis of fermented foods can yield insights into the nature of microbial interactions or microbial community formation in other, more complicated environments [37]. However, the absence of a consensus with respect to the optimal sequencing platform or bioinformatic tools for shotgun metagenomic analysis of simple microbial communities could delay the more widespread application of the approach.

Here, we describe the first comparison of the performances of the short-read DNA sequencing platforms, the Illumina MiSeq, the Illumina NextSeq, and the Ion Proton, for shotgun metagenomic analysis of low-complexity food-associated microbial communities. This analysis was combined with an investigation of the impact of sequencing depth and downstream bioinformatic analysis, with a view to informing researchers, and especially food microbiologists, when designing shotgun metagenomic experiments.

## Results

### Compositional analysis is influenced more by the choice of species classifier than the platform used

**Fig. 1:**
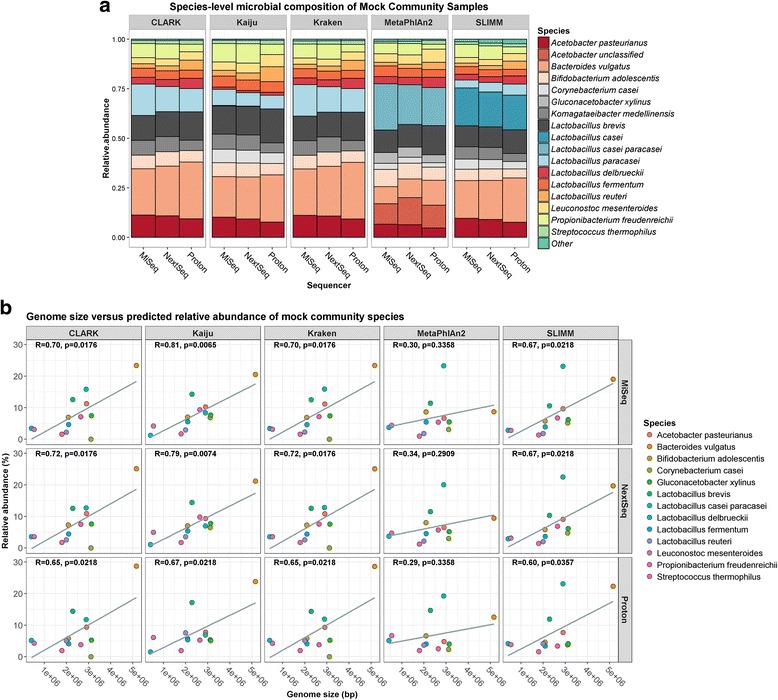
Compositional analysis of the mock community using the total number of reads from each sequencer. **a** Species-level profile of the mock community, as determined by each species classifier. **b** Correlations between the relative abundances of species with their respective genome sizes

The Illumina MiSeq, the Illumina NextSeq, and the Ion Proton platforms were used for shogun metagenomic sequencing of a mock community sample, containing an equimolar mixture of genomic DNA from 13 food-related bacteria (Table 1), as well as six kefir DNA samples. The MiSeq produced 1,869,744 ± 401,024 reads per sample. The NextSeq produced 13,415,363 ± 4,098,763 reads per sample. The Proton produced 19,328,498 ± 3,240,112 reads per sample. The species classifiers CLARK, Kaiju, Kraken, MetaPhlAn2, and SLIMM were used to determine the bacterial composition of the samples. Compositional analysis of the mock community sample were generally consistent across the three platforms (Fig. 1a), although some minor differences were observed, particularly between the Illumina sequencers versus the Ion Proton. For example, based on the average results from each species classifier, the MiSeq, the NextSeq, and the Proton detected *Acetobacter pasteurianus* in the mock community sample at 9.8, 9.3, and 7.8%, respectively, and *Lactobacillus reuteri* in the same sample at 2.2, 2.5, and 5.1%, respectively. With respect to species classifier, based on the average results from each sequencer, *Bacteroides vulgatus* was detected at 25.7% with CLARK compared to 10.2% with MetaPhlAn2, while *Lactobacillus brevis* was detected at 15.3% with Kaiju compared to 10.9% with SLIMM. Additionally, Kaiju, MetaPhlAn2, and SLIMM detected all 13 mock community species from data generated from each of the sequencing platforms used, whereas CLARK and Kraken did not detect *Corynebacterium casei* from any of the datasets, despite this species being represented with their respective databases. The mock community species were not present at equal relative abundances in any sample, despite genomic DNA having being mixed in equimolar ratios. For example, based on the average results from all data, the relative abundance of *Bacteroides vulgatus* was 20.8%, whereas the relative abundance of *Streptococcus thermophilus* was 1.6%. Indeed, the relative abundances of mock community species positively correlated with their genome size for all of the classifiers, apart from MetaPhlAn2 (Fig. 1b). However, this observation is not entirely unexpected, since it is logical that larger reference genomes will receive more hits than smaller ones, and the issue has already been reported elsewhere [38]. We subsequently found that normalising relative abundances, as predicted by CLARK, Kaiju, Kraken, and SLIMM, according to reference genome sizes resulted, on average, in a more equal distribution (Levene’s test: *p* = 0.01) (Additional file 1: Figure S1). Note that since the *L. delbrueckii* DSM 20081 and *L. mesenteroides* LMG 6909 reference genomes were incomplete (Table 1), we normalised their abundances according to the median genome size for each species.

**Table 1:** Bacterial strains whose genomic DNA was mixed in an equimolar ratio to construct the Mock Community DNA sample

Species	Strain	RefSeq assembly accession	GC content (%)	Genome size (bp)
*Acetobacter pasteurianus*	LMG 1513	GCF_000010825.1	53.1	2,907,495
*Bacteroides vulgatus*	DSM 1447	GCF_000012825.1	42.2	5,163,189
*Bifidobacterium adolescentis Reuter*	DSM 20083	GCF_000010425.1	59.3	2,089,645
*Corynebacterium casei*	LMG 19264	GCF_000550785.1	55.7	3,113,488
*Gluconacetobacter medellinensis*	LMG 1693	GCF_000182745.2	66.3	3,136,818
*Lactobacillus brevis*	ATCC 376	GCF_000014465.1	45.6	2,291,220
*Lactobacillus casei*	ATCC 334	GCF_000014525.1	46.6	2,895,264
*Lactobacillus delbrueckii*	DSM 20081*	GCF_001437195.1	49.7	415,890
*Lactobacillus fermentum*	LMG 18251	GCF_000010145.1	51.8	2,098,685
*Lactobacillus reuteri*	DSM 20016	GCF_000016825.1	38.9	1,999,618
*Leuconostoc mesenteroides*	LMG 6909*	GCF_000160595.1	37.7	543,364
*Propionibacterium freudenreichii*	LMG 16412	GCF_000940845.1	67.3	2,649,166
*Streptococcus thermophilus*	LMG 18311	GCF_000011825.1	39.1	1,796,846

*Incomplete genome sequence

A number of species not present in the mock community DNA sample were detected as false positives (Additional file 2: Figure S2). With respect to platforms, the MiSeq and NextSeq gave the lowest and highest numbers of false positives, respectively. Of the species classifiers, MetaPhlAn2 and SLIMM gave the lowest and highest numbers of false positives, respectively. However, it is important to note that all of the false positives were detected at less than 1% relative abundance, and species assigned were closely related to actual mock community species.

Overall, our results indicate that MetaPhlAn2 is the most accurate method, since it provided the lowest number of false positives. Additionally, the relative abundances predicted by MetaPhlAn2 were not biased by reference genome sizes.

**Fig. 2:**
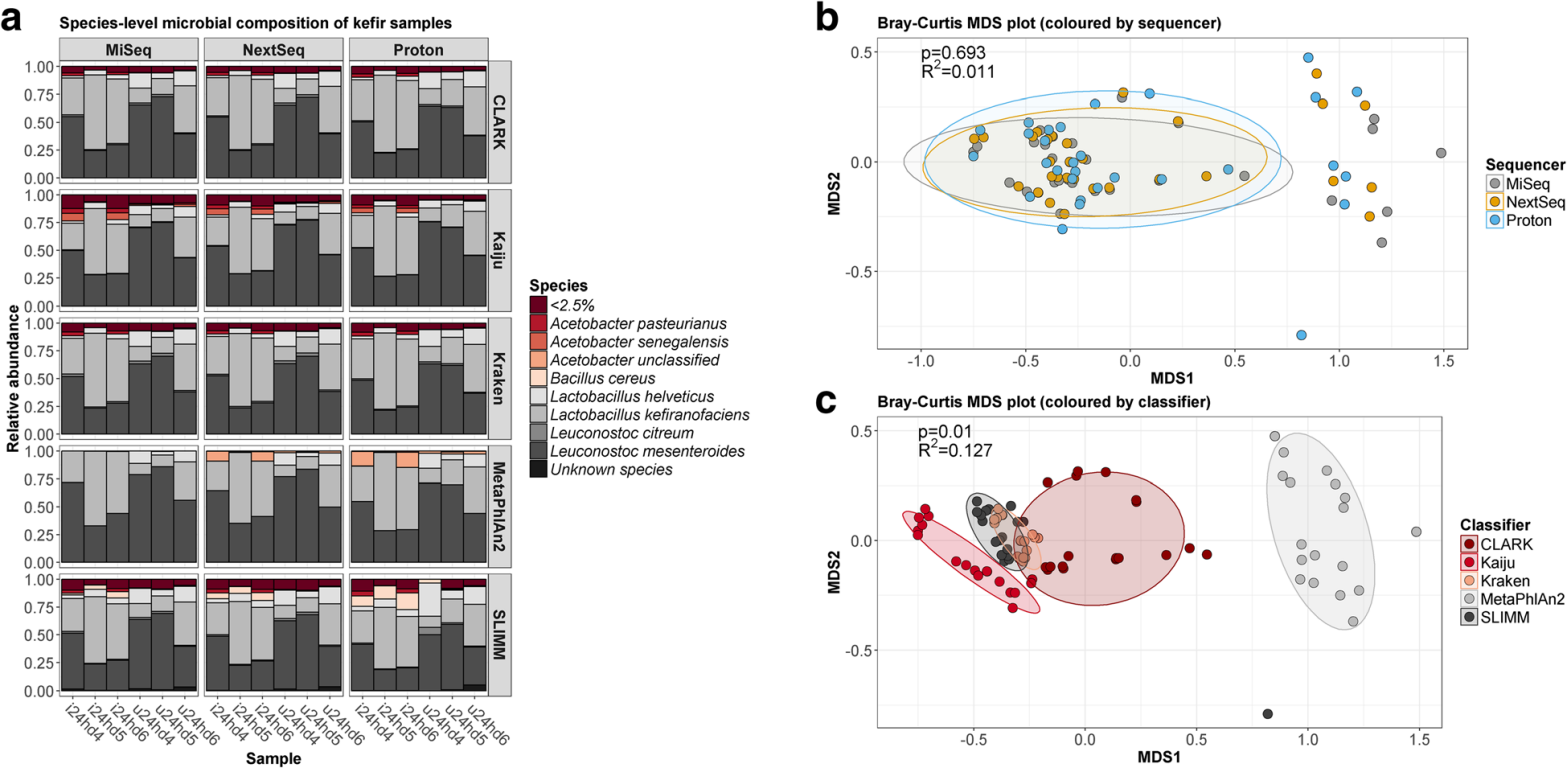
Compositional analysis of kefir samples using the total number of reads from each sequencer. **a** Species-level profile of the kefir samples, as determined by each species classifier. **b** Dissimilarity plot showing differences between sequencers. **c** Dissimilarity plot showing differences between species classifiers

The microbiota composition of kefir samples were similar as determined across the three platforms (Fig. 2a), but again, there were some significant differences. Specifically, two classifiers, Kaiju and SLIMM, indicated that *Lactobacillus plantarum* was present at significantly lower ratios in MiSeq-sequenced samples than in proton-sequenced samples (Kaiju: *p* = 0.031; SLIMM: *p* = 0.031), and SLIMM also indicated that *Lactobacillus acidophilus* was significantly lower in MiSeq samples than in NextSeq samples (*p* = 0.019). MetaPhlAn2 also failed to detect *Acetobacter* in MiSeq samples, but the tool did identify *Acetobacter* in the other sample groups. Alpha diversity measures were not significantly different between sequencers (Additional file 3: Table S1), but they were significantly different between classifiers (Additional file 4: Table S2). Specifically, the alpha diversity predicted by MetaPhlAn2 was lower than that by any other classifier, while the alpha diversity predicted by CLARK was also lower than that by SLIMM. Multidimensional scaling (MDS) analysis of compositional data confirmed that there was no significant dissimilarity between the sequencers (PERMANOVA: *p* = 0.693, *R*^2^ = 0.011) (Fig. 2b), but it revealed that there was a significant dissimilarity between the species classifiers (PERMANOVA: *p* = 0.01, *R*^2^ = 0.127) (Fig. 2c). MetaPhlAn2 was especially different from the other classifiers, since it did not detect *Acetobacter pasteurianus* or *Leuconostoc citreum* (Additional file 5: Figure S3). Thus, although the mock community analysis indicated that MetaPhlAn2 is the most accurate approach, these results suggest that it is less sensitive than the other methods. Furthermore, only Kaiju detected *Acetobacter senegalensis*, while only SLIMM detected *Bacillus cereus* (Additional file 5: Figure S3). However, there were no significant differences in the abundances of the two dominant kefir species, *Lactobacillus kefiranofaciens* or *Leuconostoc mesenteroides*, between any classifier (Additional file 6: Table S3).

We averaged the results from each species classifier to generate a consensus taxonomic profile of the kefir samples (Additional file 7: Figure S4A), and subsequent MDS analysis verified that there was no significant dissimilarity between the sequencers (PERMANOVA: *p* = 0.912, *R*^2^ = 0.02) (Additional file 7: Figure S4B).

### Bacterial strain identification was consistent across platforms

**Fig. 3:**
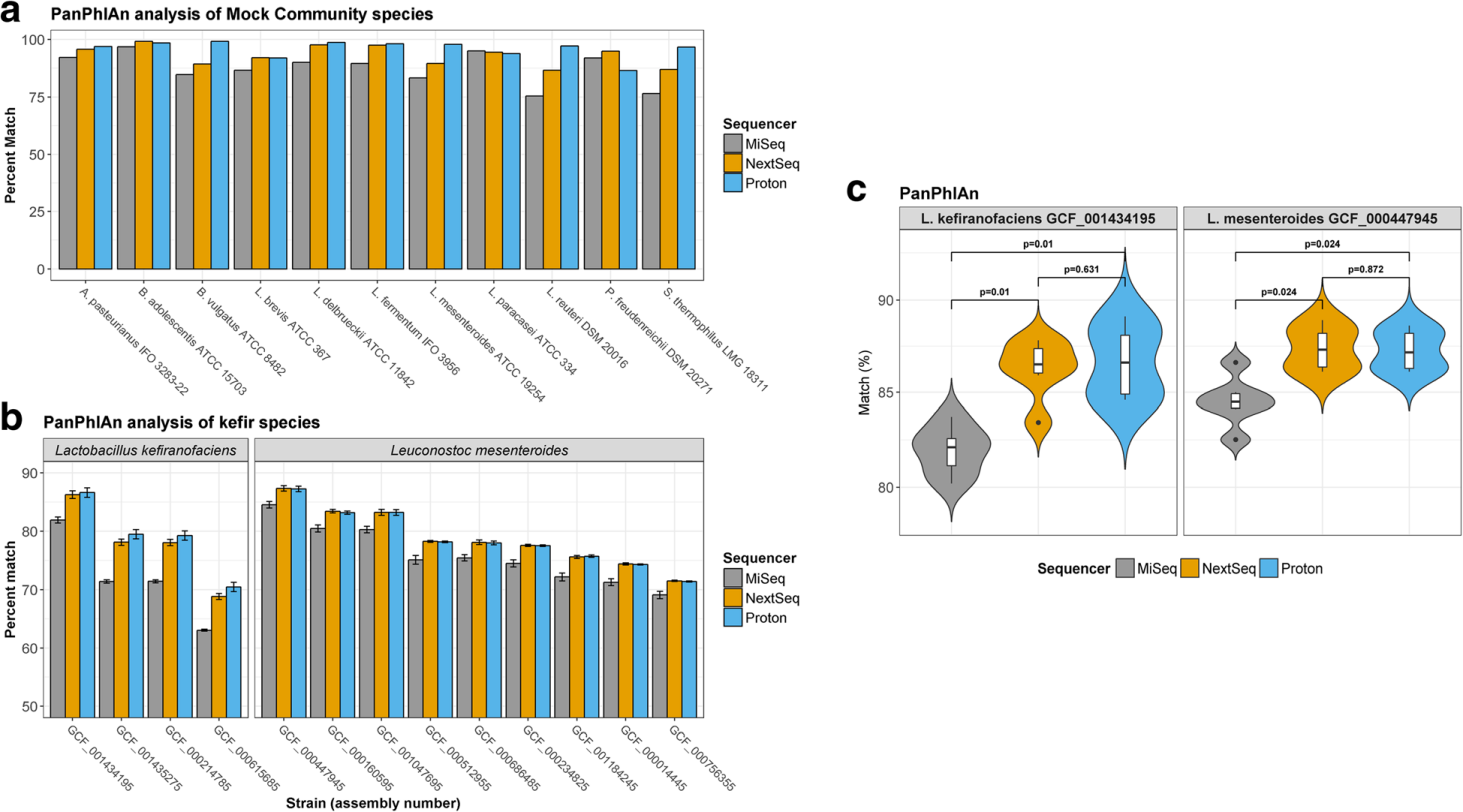
Strain-level analysis, with PanPhlAn, using the total number of reads from each sequencer. **a** The highest match for each of 11 mock community species for which ≥ 2 reference strain genomes are available at RefSeq, based on the presence/absence of pangenome gene families. **b** A comparison of the relatedness of the *Lactobacillus kefiranofaciens* and *Leuconostoc mesenteroides* strains detected in kefir samples with each of the reference strain genomes present in the respective **c** Statistical differences in the proportion of PanPhlAn pangenome gene families detected using each sequencer

To further increase taxonomic resolution, we used PanPhlAn to characterise bacterial strains present in the samples. The results of strain-level metagenomic analyses were consistent across the three sequencers. For the mock community sample, PanPhlAn identified the correct strain of each of the analysed species (Fig. 3a). For example, the MiSeq, NextSeq, and Proton indicated that the *Lactobacillus fermentum* strain in the mock community shared 89.6, 97.5, and 98.1%, respectively, of its pangenome gene families with *L. fermentum* IFO 3956, while they indicated that the *Streptococcus thermophilus* strain in the mock community shared 76.6, 86.9, and 96.7%, respectively, of its pangenome gene families with *S. thermophilus* LMG 18311. Note that greater than two reference genomes are needed to construct a PanPhlAn pangenome database, and hence, we were unable to use PanPhlAn for strain-level analysis of *Corynebacterium casei* or *Gluconacetobacter xylinus.*

For the kefir samples, PanPhlAn was used to provide strain-level analysis of the two most dominant species, *Lactobacillus kefiranofaciens* and *Leuconostoc mesenteroides*. Analysis on the MiSeq, NextSeq, and Proton platforms all indicated that the *Lactobacillus kefiranofaciens* strain detected in the kefir samples was most closely related to *L. kefiranofaciens* GCF_001434195, but the MiSeq detected significantly fewer shared pangenome gene families than either the NextSeq (*p* = 0.01) or the Proton (*p* = 0.01). Similarly, analysis of data from all the three platforms indicated that the *Leuconostoc mesenteroides* strain was most closely related to *L. mesenteroides* GCF_000447945 (Fig. 3b), but, again, the MiSeq detected significantly fewer shared pangenome gene families than either the NextSeq (*p* = 0.024) or the Proton (*p* = 0.024). It is likely that the decreased accuracy achieved with the MiSeq was due to its lower sequencing depth relative to the other two sequencers. The contribution of sequencing depth to the accuracy of strain-level analysis is investigated in the subsequent sections.

### Metagenome assembly completeness varies significantly between platforms but functional profiles remain consistent

IDBA-UD was used to assemble the mock community and kefir metagenomes. The n50 number, which is a measure of metagenome assembly completeness, of MiSeq assemblies was significantly lower than either that of NextSeq (*p* = 0.03) or Proton assemblies (*p* = 0.011) (Additional file 8: Figure S5). The mean n50 numbers for each platform were as follows: n50 = 3151 (MiSeq), n50 = 13,874 (NextSeq), and n50 = 9307 (Proton).

**Fig. 4:**
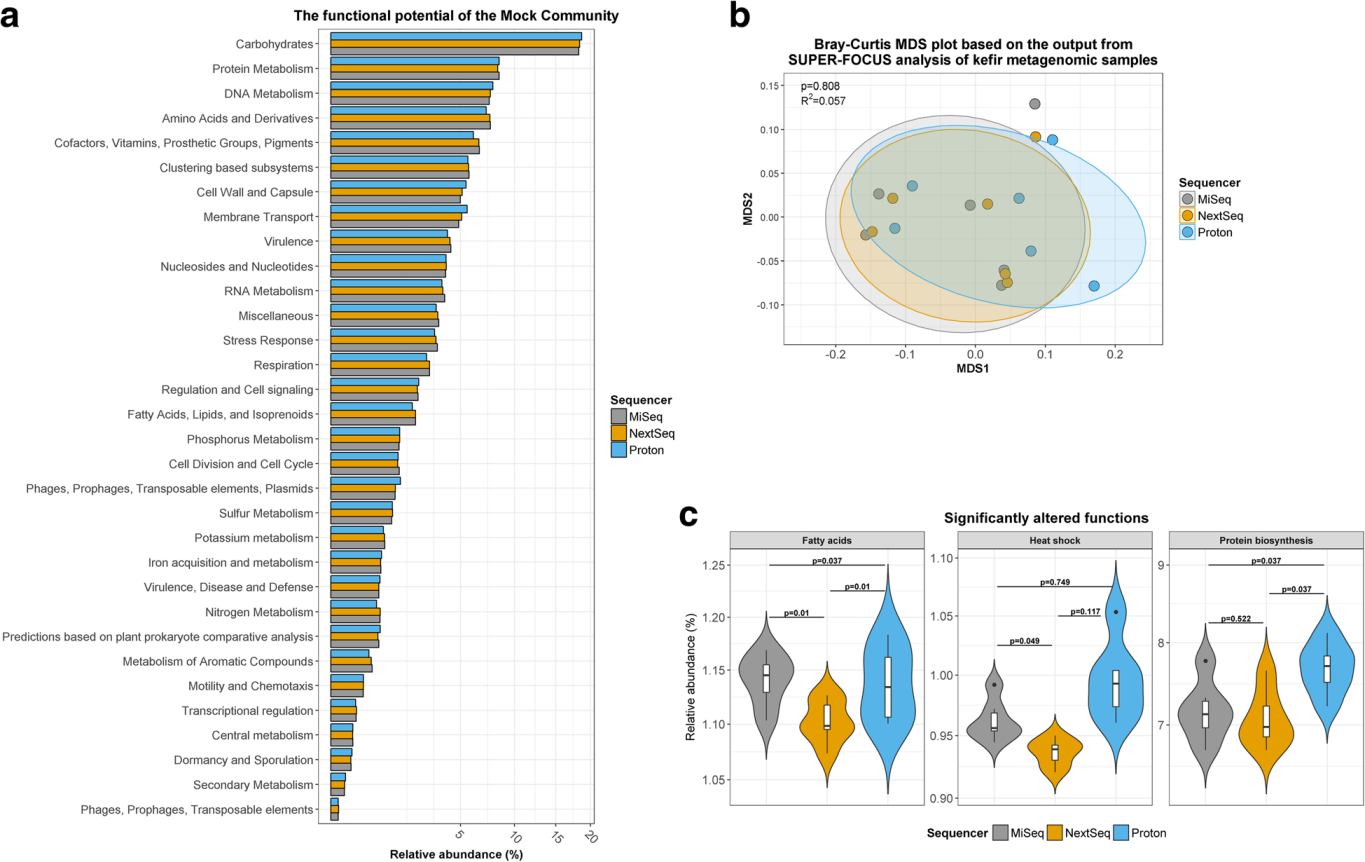
Functional analysis, with SUPER-FOCUS, using the total number of sequences from each sequencer. **a** The relative abundances of SUPER-FOCUS level 1 subsystems detected in the mock community. **b** Dissimilarity plot based on the relative abundances of the SUPER-FOCUS level 3 subsystems detected in the kefir samples. **c** SUPER-FOCUS level 2 subsystems which were significantly altered between sequencers

The functional profile of the mock community sample, as characterised by SUPER-FOCUS, was congruent across the three platforms (Fig. 4a). As anticipated, a large proportion of the metagenome was involved in housekeeping functions such as carbohydrate or protein metabolism. Specifically, the MiSeq, NextSeq, and Proton detected the “carbohydrates” subsystem at 18.2, 18.4, and 18.7%, respectively, while they detected the “protein metabolism” subsystem at 8.4%, 8.3%, and 8.4%, respectively. Similarly, the functional potential of kefir samples was accordant across the three platforms. Indeed, MDS analysis indicated that the Illumina sequencers were more similar to each other than the Proton, but there was no significant overall dissimilarity between the three sequencers (PERMANOVA: *p* = 0.808, *R*^2^ = 0.057) (Fig. 4b). However, we did observe significant differences in the abundances of three SUPER-FOCUS subsystems that were present at greater than 1% relative abundances in kefir. Specifically, assignments to the “fatty acid” subsystem were significantly higher among the samples sequenced on the MiSeq than those sequenced with the NextSeq (*p* = 0.049); levels of “heat shock” subsystem-assigned reads were significantly different between all three platforms (MiSeq versus NextSeq: *p* = 0.01; MiSeq versus Proton: *p* = 0.037; NextSeq versus Proton: *p* = 0.01); and reads assigned to the “protein biosynthesis” subsystem were significantly higher among samples sequenced on the Proton than those sequenced with either on the MiSeq (*p* = 0.037) or the NextSeq (*p* = 0.037) (Fig. 4c).

### Metagenomic pathway analysis tools provide inconsistent results

The results from SUPER-FOCUS were compared to those from HUMAnN2, which is an alternative tool for functional analysis of metagenomes. MDS analysis revealed that there was a significant dissimilarity between the two tools (PERMANOVA: *p* = 0.808, *R*^2^ = 0.057) (Additional file 9: Figure S6), based on the relative abundances of 865 level-4 enzyme commission (EC) categories which were detected by both programs. Indeed, 749 EC categories were differentially abundant between the methods (Additional file 10: Table S4).

### Sequencing depth does not significantly affect composition or functional potential of low-complexity food microbiomes

Reads from the mock community and kefir samples were randomly subsampled to assess the effects of sequencing depth on compositional and functional analysis. MiSeq reads were subsampled from 100,000 to 1,000,000 reads per sample, while NextSeq and Proton reads were subsampled from 100,000 to 7,500,000 reads per sample.

**Fig. 5:**
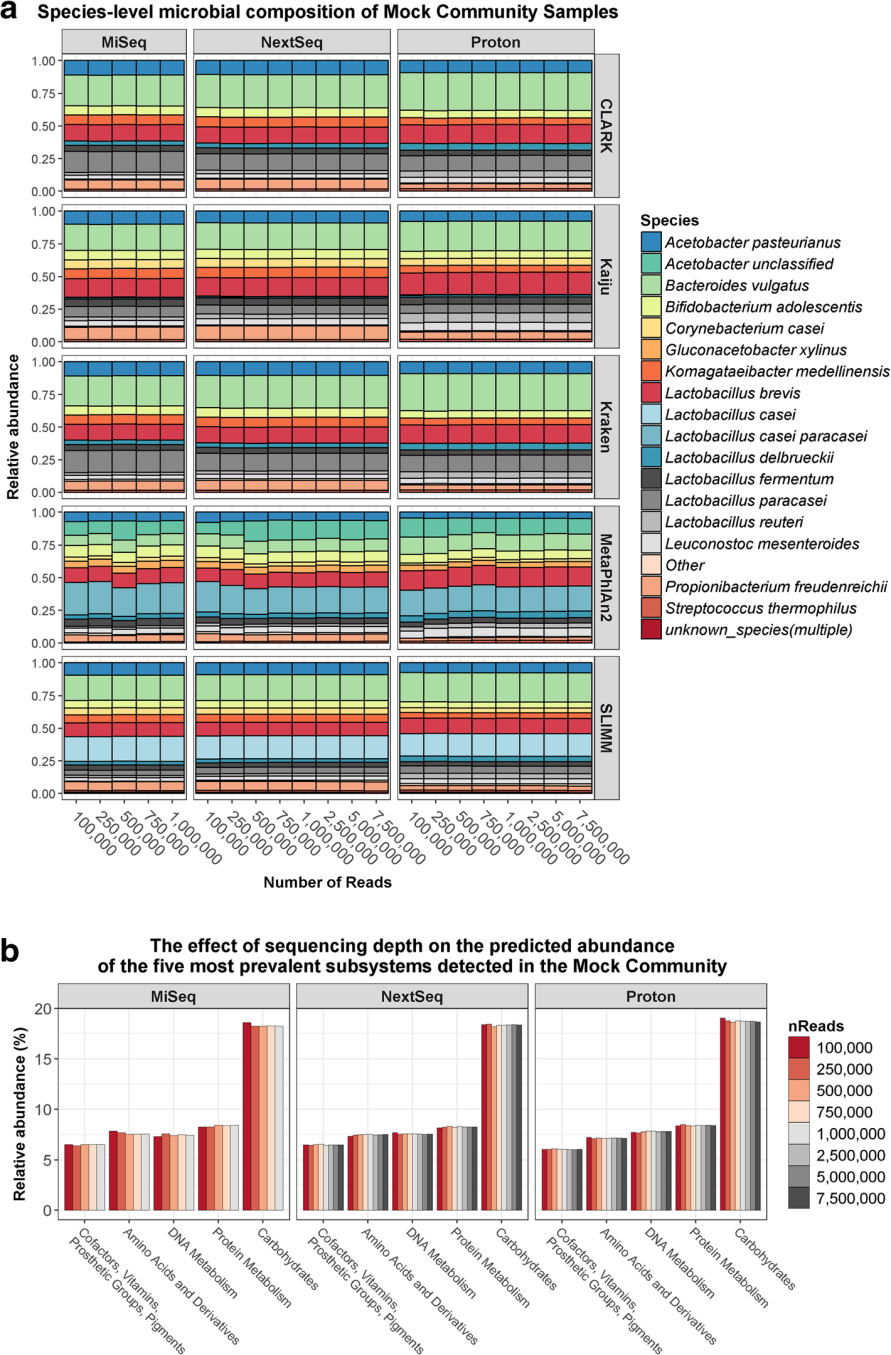
The effect of sequencing depth on compositional and functional analysis of the mock community. **a** The species-level profile of the mock community sample at different sequencing depths on each sequencer. **b** The relative abundances of the top 5 most prevalent SUPER-FOCUS level 1 subsystems detected in the mock community at different sequencing depths on each sequencer

For the mock community sample, the compositions were close to identical, regardless of sequencing depth (Fig. 5a). For example, Kraken detected *Lactobacillus reuteri* at 2.6% using 100,000 NextSeq reads, while it was detected at 2.5% using 7,500,000 NextSeq reads. Similarly, the results of compositional analysis were uniform at divergent sequencing depths (Fig. 5b). For instance, based on SUPER-FOCUS results, the carbohydrate metabolism subsystem was detected at 18.6% using 100,000 NextSeq reads, while it was detected at 18.4% using 7,500,000 NextSeq reads.

**Fig. 6:**
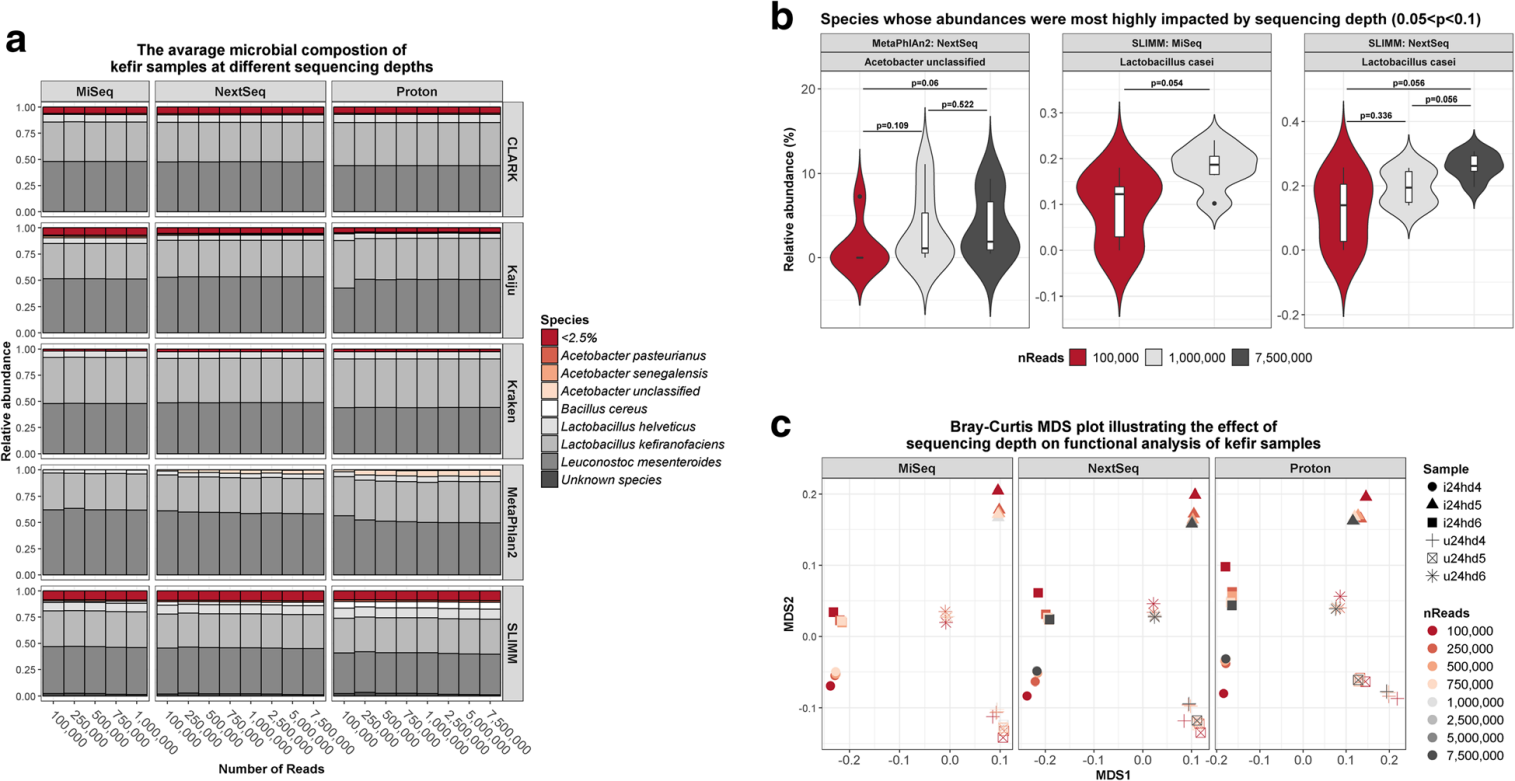
The effect of sequencing depth on compositional and functional analysis of kefir. **a** The average species-level profile of kefir samples at different sequencing depths on each sequencer. **b** Species whose abundances were most highly impacted by sequencing depth (0.05 < *p* < 0.1). **c** Dissimilarity plot based on the relative abundances of the SUPER-FOCUS level 3 subsystems detected in the kefir samples at different sequencing depths on each sequencer

The microbial profiles of the subsampled kefir reads were highly similar at different sequencing depths (Fig. 6a). Indeed, there were no significant differences in the abundances of any species present at > 0.1% relative abundance, as detected by each classifier, at sequencing depths of 100,000, 1,000,000, or 7,500,000 reads per sample. However, we did observe some notable, albeit non-significant, differences (Fig. 6b). Specifically, MetaPhlAn2 indicated that the abundance of *Acetobacter* was lower at 100,000 NextSeq reads compared to 7,500,000 NextSeq reads (*p* = 0.06). SLIMM indicated that the abundance of *Latcobacillus casei* was lower at 100,000 MiSeq reads compared to 1,000,000 MiSeq reads (*p* = 0.054); 100,000 NextSeq reads compared to 7,500,000 NextSeq reads (*p* = 0.056); and 1,000,000 NextSeq reads compared to 7,500,000 NextSeq reads (*p* = 0.056). Additionally, there were no significant differences in alpha diversity at these different sequencing depths on any sequencer (Additional file 11: Table S5), although alpha diversity measures predicted by MetaPhlAn2 did visibly increase with sequencing depths up to 1,000,000 reads per sample (Additional file 12: Figure S7A). Similarly, MDS analysis indicated that there were no clear differences in microbial composition predicted by CLARK, Kaiju, Kraken, or SLIMM at different sequencing depths, but there were apparent differences between the microbial compositions predicted by MetaPhlAn2 at different sequencing depths (Additional file 12: Figure S7B). It is important to note that we only included species which were detected at > 0.1% relative abundance in our diversity analysis. It is possible that higher sequencing depths might improve the detection of species present at < 0.1%, which may affect diversity measures.

SUPER-FOCUS analysis of subsampled kefir reads again revealed that the functional profiles were highly similar at the different sequencing depths. Indeed, MDS analysis indicated that data points did not cluster by the number of reads per sample (Fig. 6c), but instead, we identified six distinct clusters, representing each of the six kefir samples. However, we did identify 15 differentially abundant level 2 subsystems at different sequencing depths, but these functions were all present at < 0.01% relative abundance (Additional file 13: Figure S8).

**Fig. 7:**
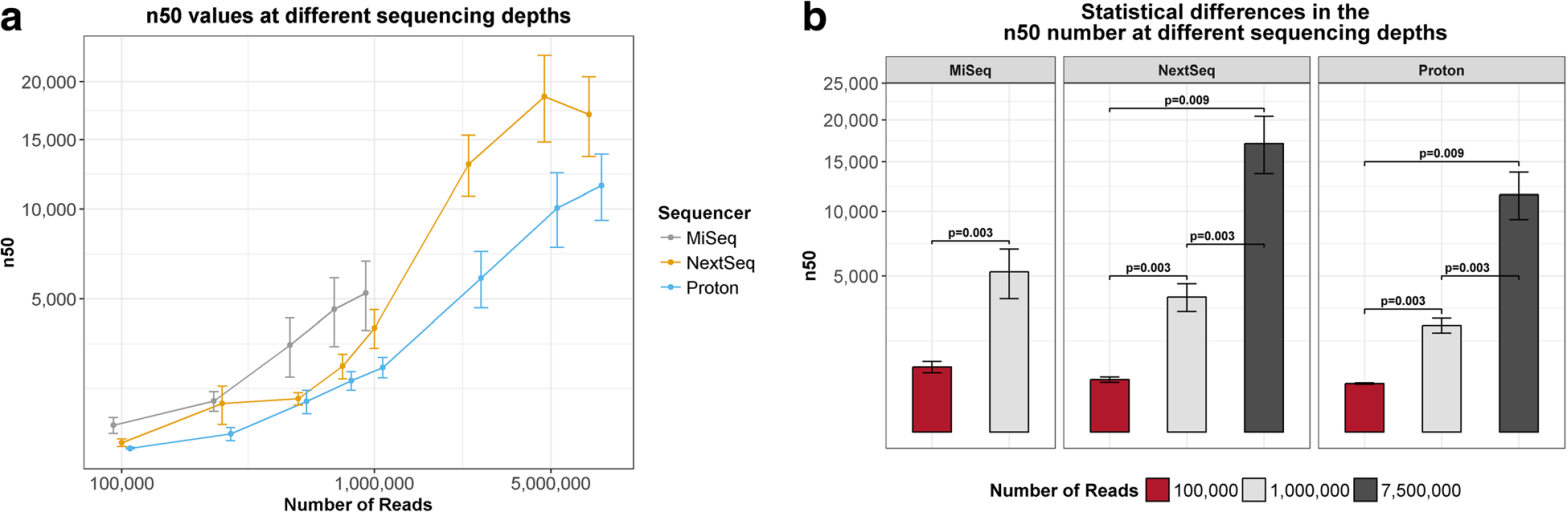
The effect of sequencing depth on metagenome assembly using IDBA-UD. **a** The n50 numbers at each sequencing depth. **b** Statistical differences in the n50 number at 100,000, 1,000,000, and 7,500,000 reads per sample

Metagenome assembly of subsampled kefir reads using IDBA-UD showed that sequencing depth had a major impact on metagenome completeness (Fig. 7a). The n50 number of metagenomes assembled from 100,000 reads was significantly lower than the n50 number of those assembled from 1,000,000 reads (*p* = 0.003) or 7,500,000 reads (*p* = 0.003) (Fig. 7b). Additionally, the n50 number of metagenomes assembled from 1,000,000 reads was significantly lower than the n50 number of those assembled from 7,500,000 reads (*p* = 0.009).

**Fig. 8:**
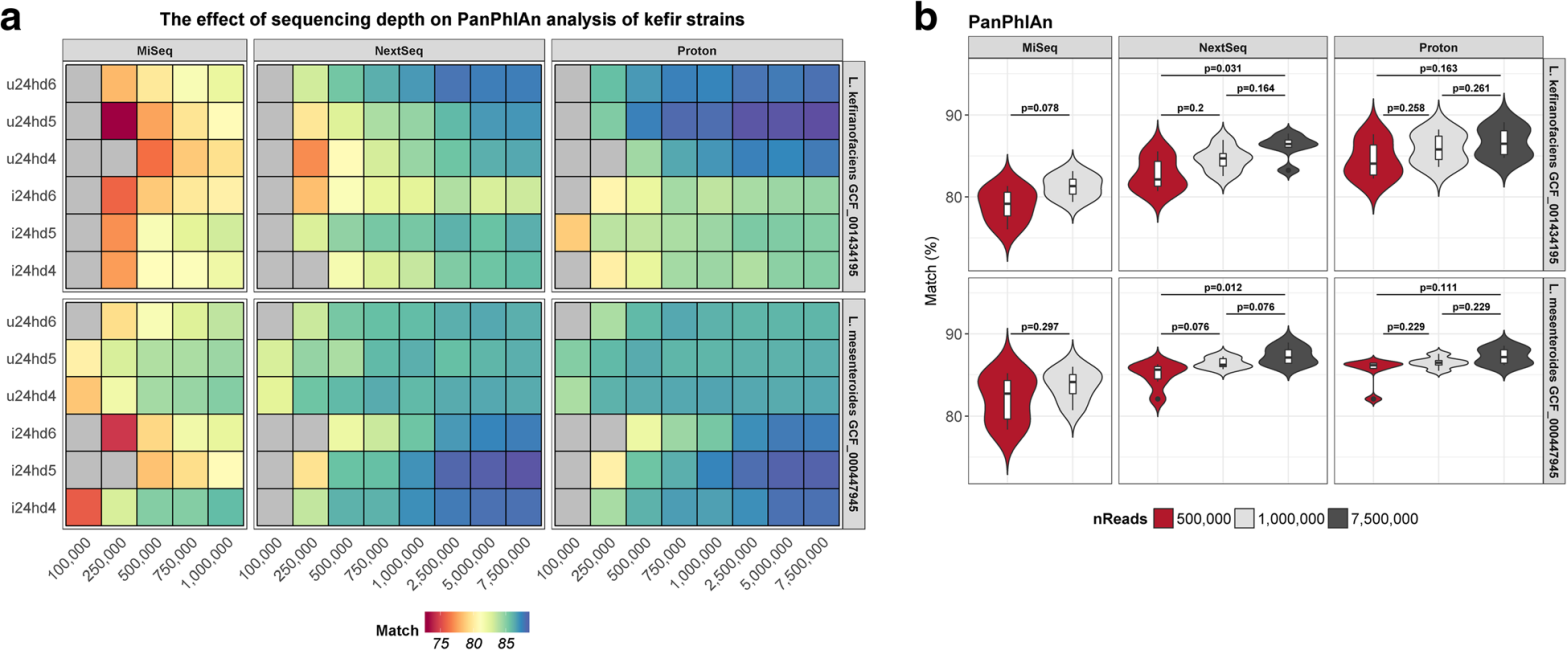
The effect of sequencing depth on PanPhlAn analysis of the two most abundant kefir species, *Lactobacillus kefiranofaciens* and *Leuconostoc mesenteroides.* **a** The predicted percentage similarity of kefir strains relative to their most closely related reference strain, at each sequencing depth. Grey cells indicate that the species was not classified to the strain level at the specified depth. **b** Statistical differences in the percentage similarity at 100,000, 1,000,000, and 7,500,000 reads per sample

Finally, we used PanPhlAn to assess the impact of sequencing depth on strain-level analysis of the two dominant kefir species, *L. kefiranofaciens* and *L. mesenteroides*. Below 500,000 reads per sample, PanPhlAn failed to characterise either species at the strain level for several kefir samples on each sequencer, but above 500,000 reads per sample, PanPhlAn successfully characterised both species at the strain level for every kefir sample on each sequencer (Fig. 8a). PanPhlAn indicated that the *L. kefiranofaciens* and *L. mesenteroides* strains detected in kefir samples shared the greatest similarity to *L. kefiranofaciens* GCF_001434195 and *L. mesenteroides* GCF_000447945, respectively. However, the percentage shared pangenome gene families was significantly lower at 500,000 reads per sample compared to 7,500,000 reads per sample on the NextSeq for both species (*L. kefiranofaciens*: *p* = 0.031; *L. mesenteroides*: *p* = 0.012) (Fig. 8b). Overall, our results indicate that the tool’s accuracy improves with increased sequencing depth.

### The reproducibility of random subsampling improves with increased sequencing depth

The reproducibility of sequence subsampling was assessed by randomly subsampling each kefir sample 10 times at 100,000 reads, 250,000 reads, and 500,000 reads. The subsampled reads were analysed using MetaPhlAn2 and SUPER-FOCUS. For MetaPhlAn2, MDS showed that replicates clustered together at each sequencing depth (Additional file 14: Figure S9A). However, the average distance from replicates to their respective centroids significantly decreased with increased sequencing depth for each sequencer (Additional file 14: Figure S9B). Additionally, at 500,000 reads, the distance to the centroid was significantly lower for the MiSeq than for either the NextSeq or the Proton (Additional file 14: Figure S9C). Similarly, for SUPER-FOCUS, MDS showed that replicates clustered together at each sequencing depth (Additional file 15: Figure S10A). However, again, the distance to the centroid significantly decreased with increased sequencing depth for each sequencer (Additional file 15: Figure S10B). Furthermore, at all sequencing depths, the distance to the centroid was lower for the MiSeq than for either the NextSeq or the Proton, and it was also lower for the NextSeq than for the Proton (Additional file 15: Figure S10C). Overall, our results indicate that random subsampling is consistent but reproducibility does improve with sequencing depth. The MiSeq gave the most consistent results, which is perhaps because it produces longer read lengths than the other two platforms.

## Discussion

Currently, there is no consensus as to which next-generation sequencing platforms are most suitable for shotgun metagenomics of low-complexity microbial communities, such as those in foods. Optimised determination of food microbiota is of considerable relevance to ensuring the safety, quality, and health-promoting attributes of foods. Here, we use a variety of bioinformatic tools to benchmark the performances of three high-throughput platforms for shotgun metagenomics of food microbial communities: the Illumina MiSeq, the Illumina NextSeq, and the Ion Proton. Our results highlight a remarkable similarity in the results generated with each platform in terms of compositional, functional, and strain-level analysis. In contrast, several issues with the outputs from species classifiers were identified. Notably, the results of MetaPhlAn2 analysis differed from those of the other species classifiers. We expect that this is because MetaPhlAn2 is based on the alignments with species-specific marker gene sequences, whereas the other methods, which can be categorised as taxonomic binning tools, are based on alignments with whole genome sequences. In fact, we noted that the relative abundances of mock community species, as predicted by all of the species classifiers apart from MetaPhlAn2, correlated to the size of their respective reference genomes. Thus, our results confirm previous observations that these species classifiers are biased by the size of the reference genome [38], in the same way that 16S rRNA gene sequencing is biased by the number of 16S rRNA genes per genome. It is important to be aware of this issue when reporting species abundances. A potential solution to the problem is to normalise relative abundances by genome size. Indeed, this solution has already been suggested elsewhere [38, 39], and we found that normalisation resulted in a more even species distribution. However, this solution is limited by the assumption that intraspecific strains share the same genome sizes, when, in fact, genome sizes often vary within a species [40]. We noted some additional discrepancies between the species classifiers. Specifically, *Corynebacterium casei* was overlooked within the mock community by CLARK or Kraken, even though the species was present in their respective databases. Compositional analysis of the mock community also produced numerous probable false positive species classifications, especially in the case of SLIMM, but most of the false positives were closely related to the actual mock community species and they were present at less than 1% relative abundance. Overall, our results indicated that none of the classifiers are entirely accurate, but we suggest that MetaPhlAn2, and perhaps Kaiju, are the most suitable for compositional analysis of low-complexity communities, especially foods, since both tools identified all of the mock community species and they can additionally detect eukaryotic organisms.

Compositional analysis of kefir showed that the choice of sequencing platform did not noticeably affect the results. However, dissimilarity analysis again highlighted marked differences between the outputs generated by the species classifiers. Thus, for compositional analysis, the choice of sequencing platform had less of an influence on results than the choice of species classifier. These observations are consistent with the findings from a previous sequencing platform comparison study [34], where the authors demonstrated that gut metagenome samples clustered by species classifier. Such results highlight a need for consistency in bioinformatics methodologies across studies, but the issue is confounded by the increasing availability of different species classifiers. The recently developed method MetaMeta [39], which integrates the results from multiple species classifiers to mitigate the flaws from each individual tool, might partially address this problem. We did not use MetaMeta here because the default program employs a different combination of species classifiers to that used in our study. Instead, we averaged the predicted taxonomic profiles from each species classifier for every sample, as an alternative solution, and subsequent analysis confirmed that there was no significant dissimilarity between the sequencers. Another possible option for compositional analysis, which we did not explore here, is to use a de novo metagenome assembly approach, wherein genomes are binned using tools like CONCOCT [41] or MetaBAT [42], and reads are then mapped against these bins to calculate species abundances. An advantage of such an approach is that it does not rely on a reference database for diversity analysis and it may also be able to estimate the abundances of potentially novel genomes. However, sequence alignment against a reference database is still necessary to assign taxonomy to the bins, and, additionally, the approach requires a considerably higher sequencing depth than short-read alignment-based methods [43].

Another important aspect of shotgun metagenomics is its ability to characterise the functional potential of metagenomes. Again, the results of functional analysis were generally consistent between all three sequencing platforms, but SUPER-FOCUS did detect significant differences in three functions which were present at greater than 1% relative abundance within the kefir metagenome. Such discrepancies suggest that results generated with different sequencers cannot be reliably compared.

Above, we described a considerable difference in the compositional profiles determined by different species classifiers. Hence, we also compared results from SUPER-FOCUS with those from HUMAnN2, which is an alternative tool for functional analysis of metagenomes. We observed a similarly pronounced disparity in the results generated by these methods. Specifically, 865 level-4 enzyme commission (EC) categories were detected with both tools, but 749 of these EC categories were differentially abundant between them. Our observation is not unexpected since these pipelines use inherently different approaches, but it does further emphasise that results obtained using different methods cannot be directly compared.

Next, we compared the results of strain-level analysis using PanPhlAn, and we found that all three sequencers correctly identified the analysed strains from the mock community sample. Similarly, the three platforms each indicated that the *L. kefiranofaciens* and *L. mesenteroides* strains detected in the kefir samples were most closely related to *L. kefiranofaciens* GCF_001434195 and *L. mesenteroides* GCF_000447945, respectively. PanPhlAn was significantly less accurate when utilising data generated by the MiSeq compared to either NextSeq or Proton data, suggesting that sequencing depth affected strain-level analysis. We subsequently confirmed this by randomly subsampling kefir sequencing reads which demonstrated that PanPhlAn failed to detect *L. kefiranofaciens* GCF_001434195 or *L. mesenteroides* GCF_000447945 a subset of kefir samples below 500,000 reads per sample using any sequencer. Similarly, and as expected, we observed that sequencing depth significantly improved metagenome assembly completeness. On the other hand, sequencing depth did not have a noticeable effect on compositional or functional analysis of the mock community or kefir, regardless of the choice of sequencer. Indeed, the results of these analyses were almost uniform at sequencing depths ranging from 100,000 reads per sample to 7,500,000 reads per sample, regardless of the choice of species classifier. It is important to note, however, that increased sequencing depth caused a slight, but significant, improvement in the reproducibility of random subsampling, which suggests that higher coverage offers more reproducible results.

Overall, our findings confirm that the Proton is on par with Illumina sequencers in terms of accuracy, but only a handful of studies have used the Proton for shotgun metagenomics to date [44, 45], even if it is widely used for human exome sequencing. On the basis of these investigations, the Proton is a viable option for metagenomic analyses.

To date, most high-throughput sequencing-based studies of microbial communities of food have relied upon 16S rRNA gene sequencing [35]. Shotgun metagenomics can, in general, offer higher taxonomic resolution than amplicon sequencing, although the latter approach may be superior for studying poorly microbiologically characterised environments that contain few species for which there are available reference genomes. Shotgun metagenomics can also be used for the direct functional characterisation of metagenomes. Several recent studies have demonstrated the enormous potential for shotgun metagenomic analysis of foods, and indeed, we have previously used the approach to identify the cause of a pink discoloration defect in Swiss-type cheeses [46], link microbial species with distinct flavours during kefir fermentation [47], and identify pathogenic strains in nunu [48]. However, the higher cost of shotgun metagenomics is considered prohibitive for commercial application of the technology by the food industry and, consequently, the approach has been relatively underutilised. This is partially due to a perception that shotgun metagenomics requires considerable sequencing depth per sample. Notably, our results suggest that this is not necessarily true for the low-complexity microbial communities present in foods and suggest that 750,000 to 1,000,000 reads per sample is sufficient for compositional and/or functional analysis of such simple communities.

## Conclusion

In conclusion, analysis of low-diversity metagenomic DNA representative of food microbial communities highlighted that outputs were consistent across a variety of sequencing platforms at different sequencing depths, but there were clear disparities between the outputs of bioinformatic tools. Thus, the choice of sequencer for shotgun metagenomics can be dictated by logistical factors, like platform availability, budget, or sample size, rather than sequencing chemistry. It is hoped that this work will guide researchers, particularly food microbiologists, in designing shotgun metagenomic experiments to exploit the extensive possibilities offered by the approach.

## Methods

### Sources of metagenomic DNA

Metagenomic DNA representative of a low-complexity, food-based, microbial community was generated by mixing equimolar ratios of genomic DNA from 13 food-related bacteria (Table 1). Strains were selected on the basis of the availability of corresponding complete or near-complete genome sequences from RefSeq [49]. Genomic DNA was sourced from ATCC, DSM, and LMG. Genomic DNA concentration was determined prior to pooling using the Qubit High Sensitivity DNA assay (BioSciences, Dublin, Ireland). We also analysed metagenomic DNA from six kefir milk samples which were previously isolated by Walsh et al. [47]. Briefly, the samples were produced using either the Ick grain (samples i24hd4, i24hd5, i24hd6) or the UK3 grain (samples u24hd4, u24hd5, u24hd6). Three separate kefir fermentations were done using each grain. Fermented kefir samples were collected after 24 h fermentation.

### DNA sequencing

Illumina libraries were prepared using the Nextera XT kit in accordance with the Nextera XT DNA Library Preparation Guide from Illumina. MiSeq libraries were sequenced on the Illumina MiSeq sequencing platform in the Teagasc sequencing facility, using a 2 × 300 cycle v3 kit, following standard Illumina sequencing protocols. NextSeq libraries were sequenced on the Illumina NextSeq 500, with a NextSeq 500/550 High Output Reagent Kit v2 (300 cycles), in accordance with the standard Illumina sequencing protocols. Proton libraries were prepared in accordance with the Ion Xpress Plus gDNA Fragment Library Preparation User Guide. Proton libraries were enriched using the ION Proton PI template OT2 200 Kit v3, and sequenced using the Ion PI Sequencing 200 Kit v3, in accordance with the standard Ion protocols.

### Bioinformatic analysis

Raw shotgun metagenomic fastq files were converted to bam files using SAMtools [50], and duplicate reads were subsequently removed using Picard Tools (https://github.com/broadinstitute/picard). Next, low-quality reads were removed using SAMtools in combination with Picard Tools. Illumina reads were filtered to 200 bp, and reads with a quality score less than Q30 were discarded. Ion Proton reads were filtered to 110 bp, and reads with a quality score less than Q20 were discarded. Processed bam files were converted to fastq files using the fastq-dump option from the NCBI SRA Toolkit (https://github.com/ncbi/sratoolkit), which were then converted to fasta files using the fq2fa option from IDBA-UD [51]. Reads were randomly subsampled using seqtk (https://github.com/lh3/seqtk).

Compositional analysis was performed using the following species classifiers: CLARK [52], Kaiju [53], Kraken [54], MetaPhlAn2 [55], and SLIMM [56]. Species detected below 0.1% relative abundance were categorised as “other” for each classifier. Note that Bowtie 2 [57] was used to map reads against the slimmDB_5k database. Strain-level metagenomic analysis was performed using PanPhlAn [12], which aligns reads against a pangenome database to functionally characterise strains. See Additional file 16 for a detailed description of the settings used for each species classifier and/or PanPhlAn. Functional analysis was performed with SUPER-FOCUS [58], using the aligner DIAMOND [59], and HUMAnN2 [60], using the --bypass-translated-search option. Briefly, SUPER-FOCUS measures the abundances of subsystems, or groups of proteins with shared functionality, by aligning sequencing reads against a reduced SEED database [61], whereas HUMAnN2 measures the abundances of UniRef clusters [62] by aligning sequences against the ChocoPhlAn database. HUMAnN2 gene families were mapped to level-4 enzyme commission (EC) categories using HUMAnN2 utility mapping files. Metagenome assembly was performed using IDBA-UD [51].

Sequence data have been deposited in the European Nucleotide Archive (ENA) under the project accession number PRJEB22610.

### Statistical analysis

Statistical analysis was performed in R-3.2.2 [63]. The vegan package (version 2.3.0) [64] was used for alpha diversity analysis, as well as Bray-Curtis-based multidimensional scaling (MDS) analysis. The adonis function in vegan was used for PERMANOVA (permutational analysis of variance) analysis, and the betadisper function, also in vegan, was used to calculate the distance of points from the centroid. The Kruskal-Wallis test was used to identify significant differences, and the resultant *p* values were adjusted using the Benjamini-Hochberg method. The Hmisc package (version 3.16.0) [65] was used for correlation analysis. The ggplot2 package (version 2.2.1) [66] was used for data visualisation.

It is important to note that the mock community DNA sample was only sequenced once on each platform, and thus, we were unable to assess technical variation across sequencing runs. However, previous studies have already demonstrated that such variation is small, accounting for 1.3 to 2.3% variation between KEGG functional profiles [67]. Additionally, we chose 0.1% relative abundance as an arbitrary cut-off to compare species or pathways, whereas, in reality, potentially important taxa or functions may be present below this threshold.

## Additional files

Additional file 1: **Figure S1.** The effect of normalising predicted relative abundances by reference genome size. The histogram shows the distribution of the relative abundances of the mock community species, before and after normalisation. The results are averaged across sequencers and metagenome binning tools (i.e. CLARK, Kaiju, Kraken, and SLIMM). (PNG 174 kb)

Additional file 2: **Figure S2.** False positives detected using each species classifier with the total number of reads from each sequencer. (PNG 128 kb)

Additional file 3: **Table S1.** Statistical differences in the alpha diversity of kefir samples between the three sequencers. (DOCX 16 kb)

Additional file 4: **Table S2.** Statistical differences in the alpha diversity of kefir samples between species classifiers. (DOCX 16 kb)

Additional file 5: **Figure S3.** Species detected ≥ 2.5% relative abundance in kefir samples using each species classifier with the total number of reads from each sequencer. (PNG 96 kb)

Additional file 6: **Table S3.** Statistical differences in the predicted species relative abundances between classifiers. (DOCX 29 kb)

Additional file 7: **Figure S4.** (A) The consensus taxonomic profile of kefir samples, as predicted by averaging the results from each species classifier. (B) Dissimilarity plot based on the average results from each species classifier. (PNG 97 kb)

Additional file 8: **Figure S5.** n50 number of metagenome assemblies which were assembled using the total number of reads from each sequencer. (PNG 24 kb)

Additional file 9: **Figure S6.** Dissimilarity plot based on the relative abundances of the 865 level-4 enzyme commission (EC) categories which were detected by both HUMAnN2 and SUPER-FOCUS. (PNG 47 kb)

Additional file 10: **Table S4.** Statistical differences in alpha diversity at different sequencing depths. (XLSX 10 kb)

Additional file 11: **Table S5.** Statistical differences in the relative abundances of enzyme commission (EC) level-4 categories between HUMAnN2 and SUPER-FOCUS. (CSV 16 kb)

Additional file 12: **Figure S7.** The effect of subsampling on the predicted diversity of kefir samples. (A) The alpha diversity of kefir samples at different sequencing depths on each sequencer. (B) Dissimilarity plot based on the relative abundances of the compositional analysis of subsampled kefir reads from each sequencer. (PNG 305 kb)

Additional file 13: **Figure S8.** SUPER-FOCUS level 2 subsystems which were significantly altered at different sequencing depths. (PNG 153 kb)

Additional file 14: **Figure S9.** Consistency in the MetaPhlAn2 profiles of randomly subsampled replicates from the same samples. (A) MDS plot (facetted by number of reads) where replicates (coloured by sample) are connected to their respective centroids. (B) The average distance of replicates to their respective centroids at each sequencing depth. (C) The average distance of replicates to their respective centroids for each sequencer. (PNG 214 kb)

Additional file 15: **Figure S10.** Consistency in the SUPER-FOCUS profiles of randomly subsampled replicates of the same samples. (A) MDS plot (facetted by number of reads) where replicates (coloured by sample) are connected to their respective centroids. (B) The average distance of replicates to their respective centroids at each sequencing depth. (C) The average distance of replicates to their respective centroids for each sequencer. (PNG 202 kb)

Additional file 16: The settings used for each species classifier and PanPhlAn. (DOCX 19 kb)
