# Supplementary figures and images for: Species classifier choice is a key consideration when analysing low-complexity food microbiome data
Source: Microbiome. 2018 Mar 20;6:50. doi: 10.1186/s40168-018-0437-0 (PMC5859664; doi:10.1186/s40168-018-0437-0)

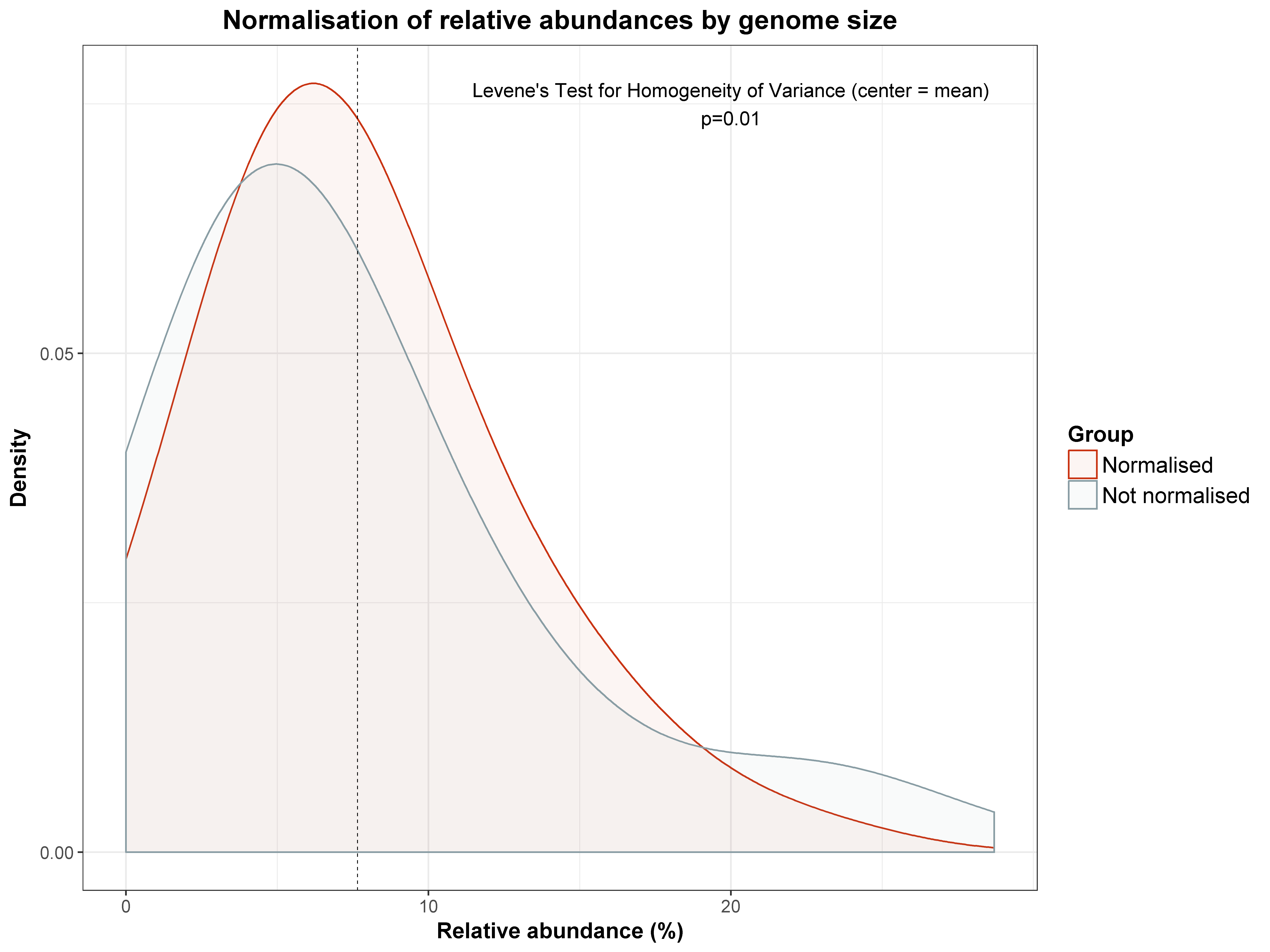

Supplement: Supplementary file 1 — Figure S1. The effect of normalising predicted relative abundances by reference genome size. The histogram shows the distribution of the relative abundances of the mock community species, before and after normalisation. The results are averaged across sequencers and metagenome binning tools (i.e. CLARK, Kaiju, Kraken, and SLIMM). (PNG 174 kb) [file 40168_2018_437_MOESM1_ESM.png]

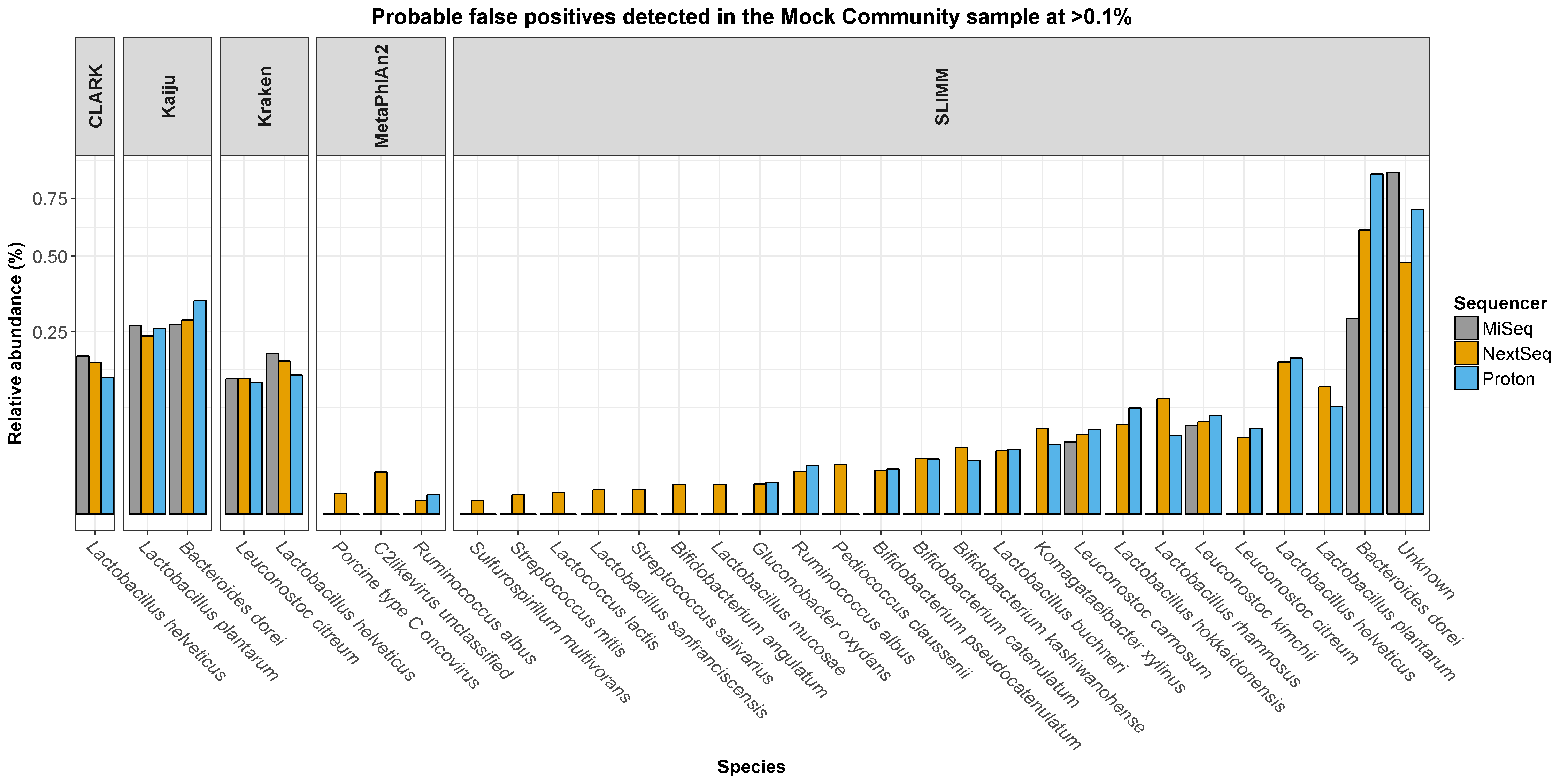

Supplement: Supplementary file 2 — Figure S2. False positives detected using each species classifier with the total number of reads from each sequencer. (PNG 128 kb) [file 40168_2018_437_MOESM2_ESM.png]

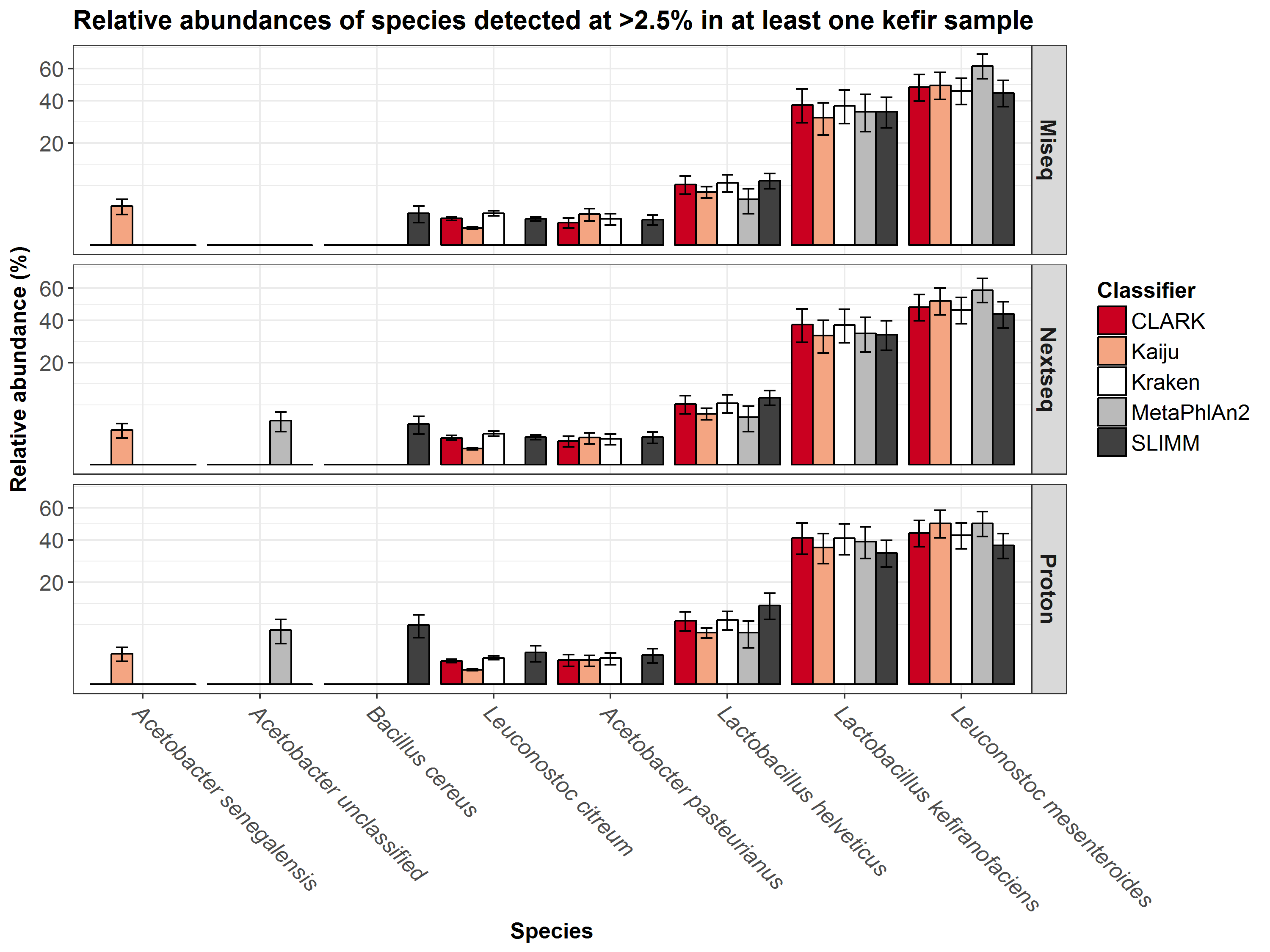

Supplement: Supplementary file 5 — Figure S3. Species detected ≥ 2.5% relative abundance in kefir samples using each species classifier with the total number of reads from each sequencer. (PNG 96 kb) [file 40168_2018_437_MOESM5_ESM.png]

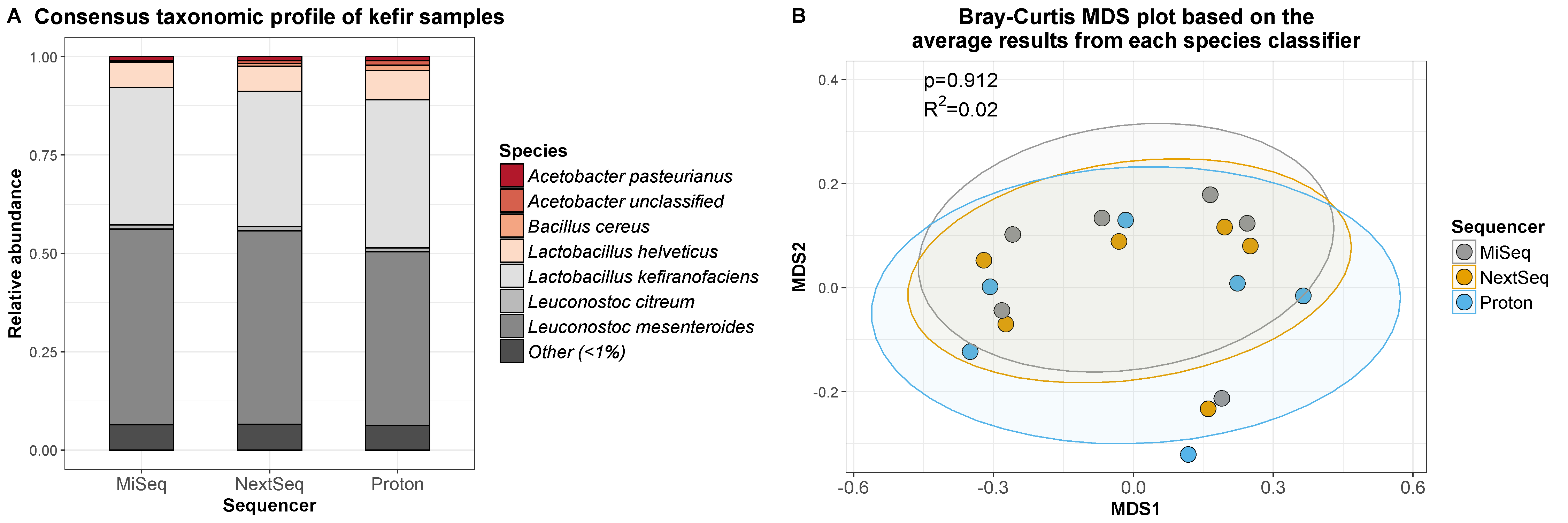

Supplement: Supplementary file 7 — Figure S4. (A) The consensus taxonomic profile of kefir samples, as predicted by averaging the results from each species classifier. (B) Dissimilarity plot based on the average results from each species classifier. (PNG 97 kb) [file 40168_2018_437_MOESM7_ESM.png]

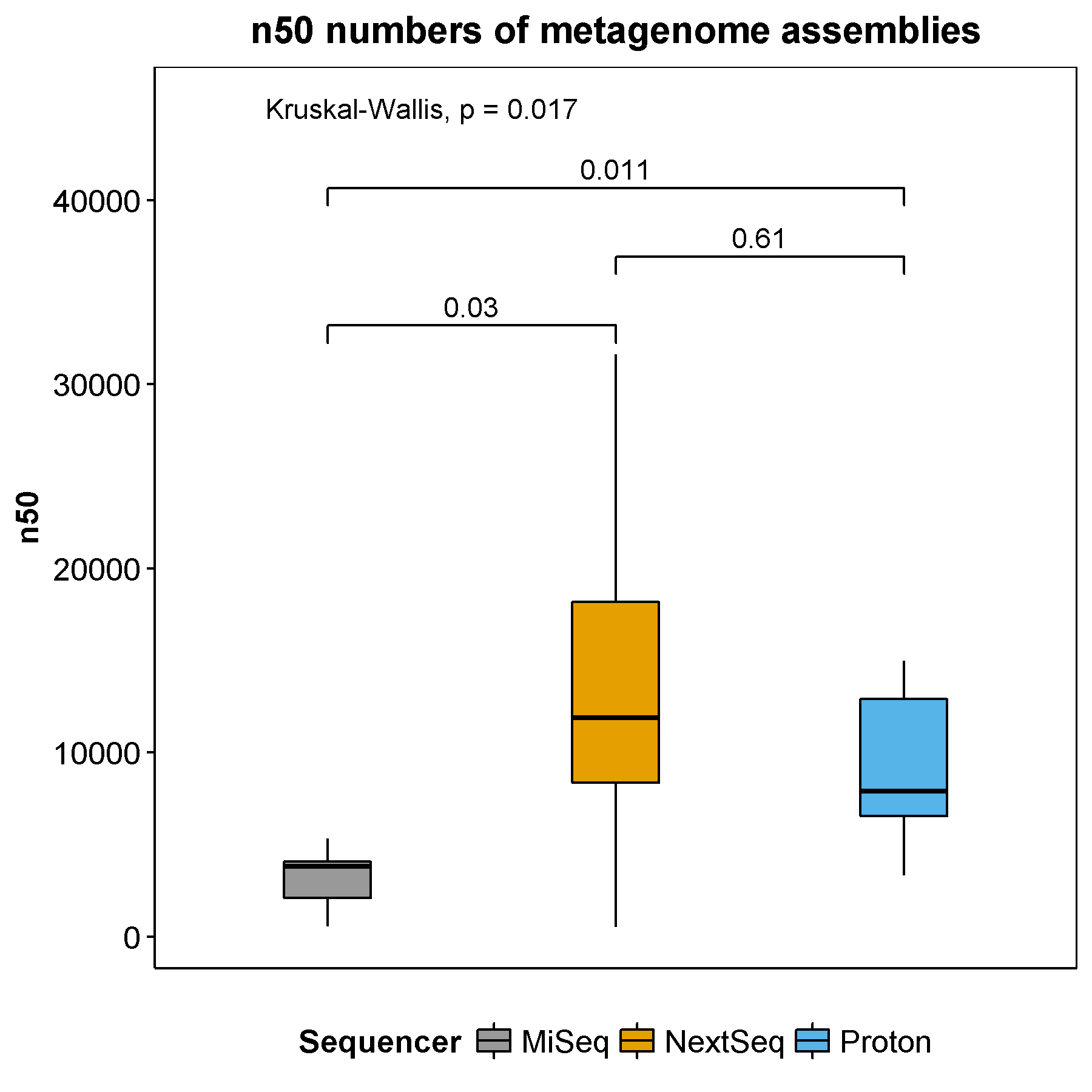

Supplement: Supplementary file 8 — Figure S5. n50 number of metagenome assemblies which were assembled using the total number of reads from each sequencer. (PNG 24 kb) [file 40168_2018_437_MOESM8_ESM.png]

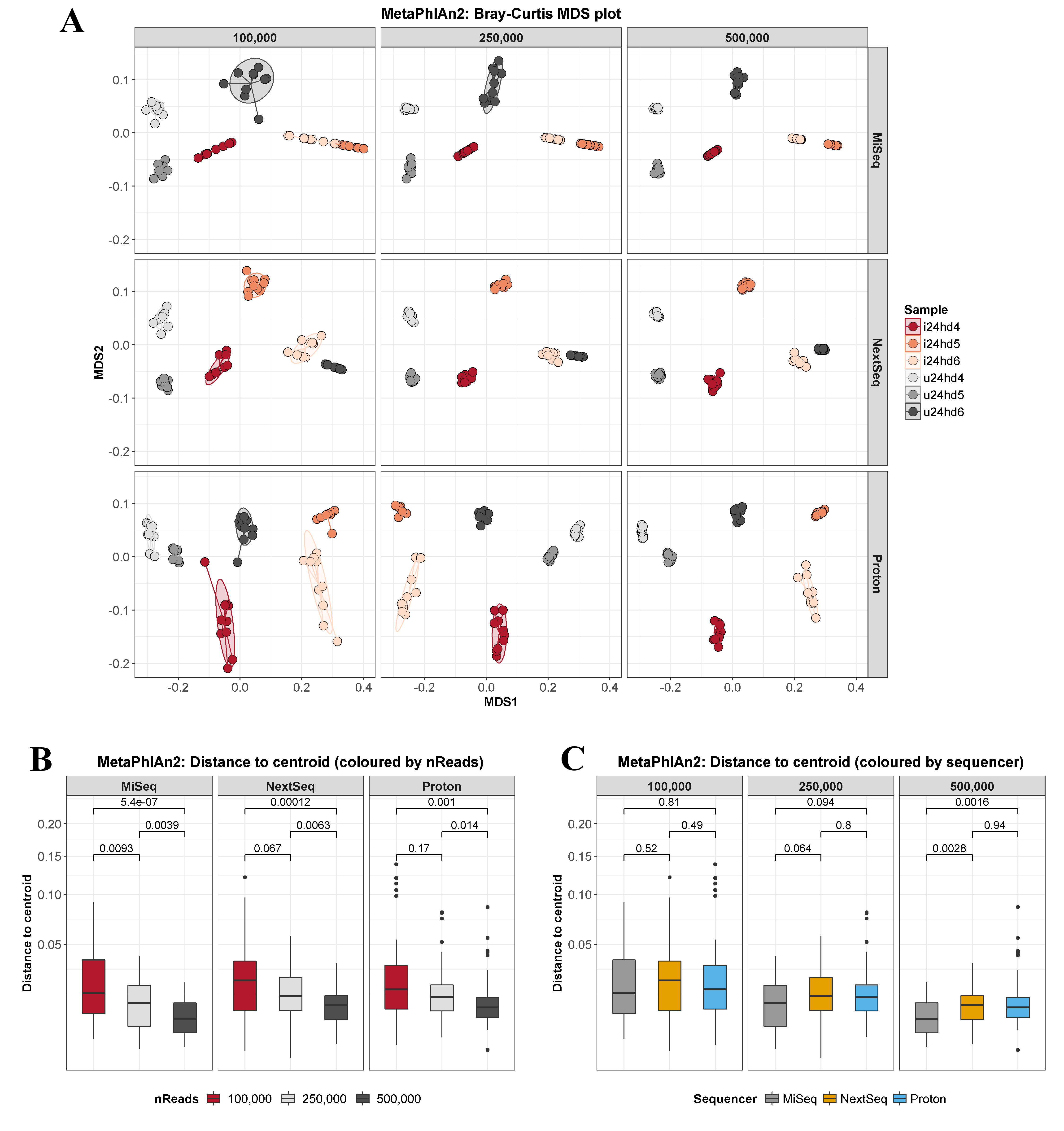

Supplement: Supplementary file 9 — Figure S6. Dissimilarity plot based on the relative abundances of the 865 level-4 enzyme commission (EC) categories which were detected by both HUMAnN2 and SUPER-FOCUS. (PNG 47 kb) [file 40168_2018_437_MOESM9_ESM.png]

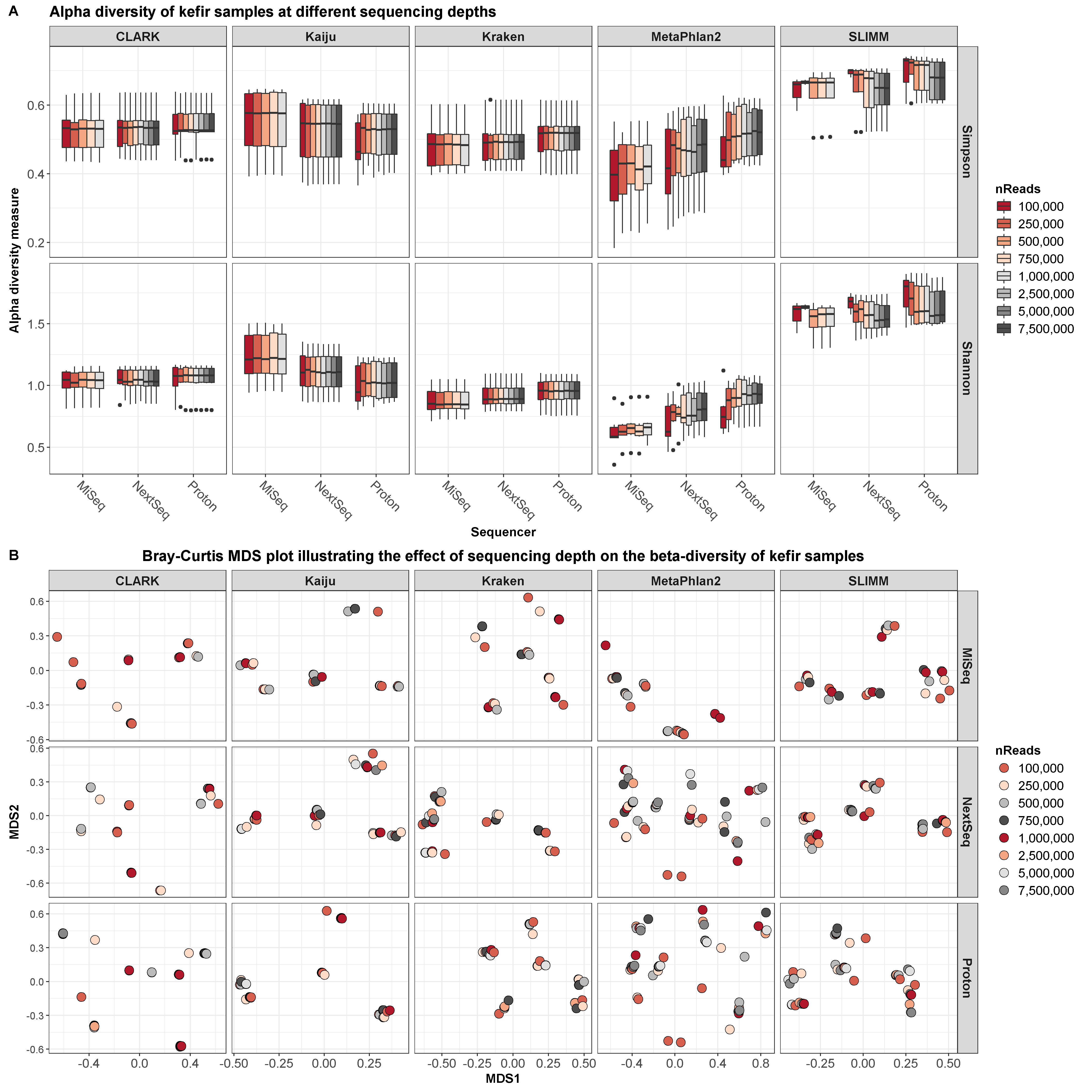

Supplement: Supplementary file 12 — Figure S7. The effect of subsampling on the predicted diversity of kefir samples. (A) The alpha diversity of kefir samples at different sequencing depths on each sequencer. (B) Dissimilarity plot based on the relative abundances of the compositional analysis of subsampled kefir reads from each sequencer. (PNG 305 kb) [file 40168_2018_437_MOESM12_ESM.png]

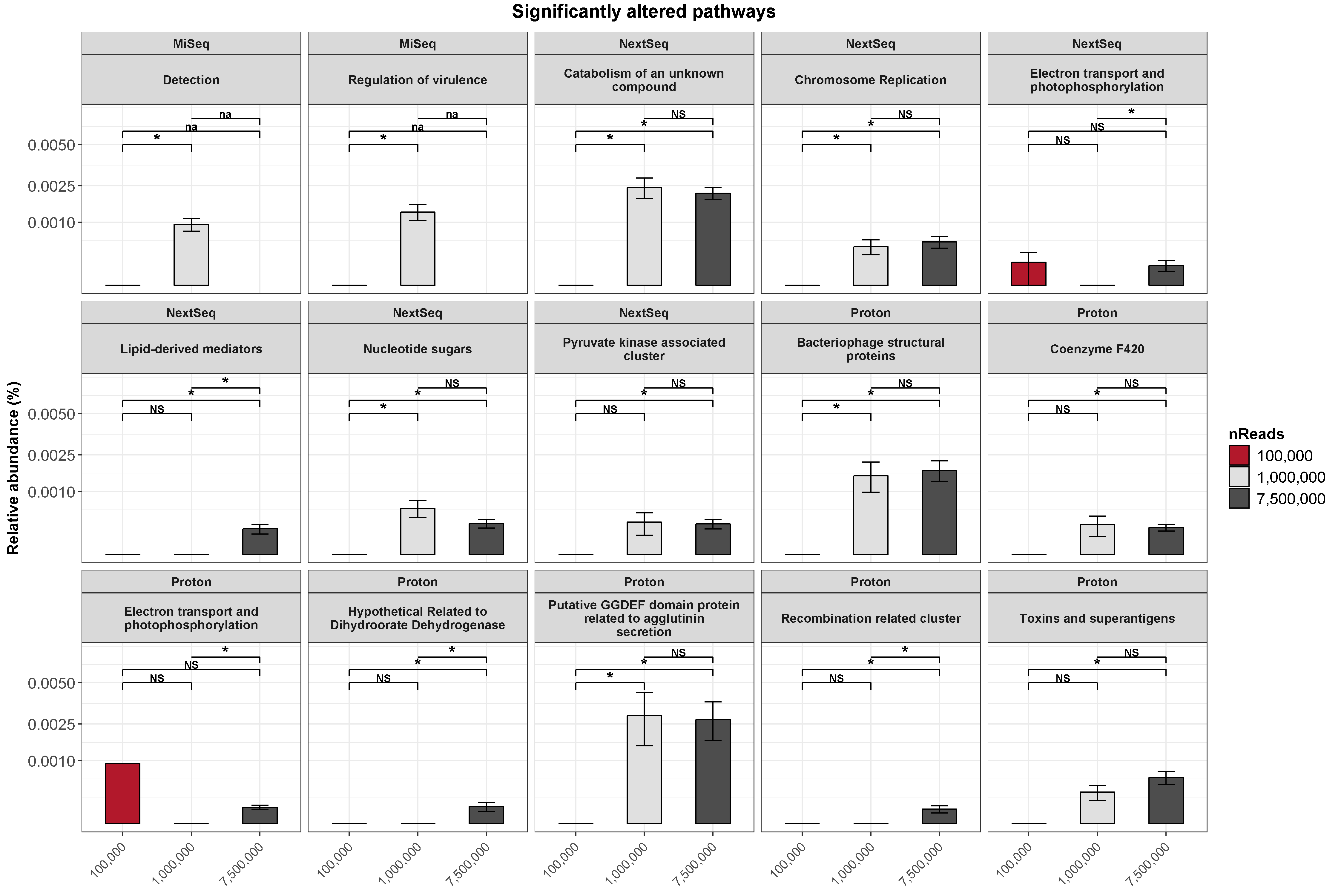

Supplement: Supplementary file 13 — Figure S8. SUPER-FOCUS level 2 subsystems which were significantly altered at different sequencing depths. (PNG 153 kb) [file 40168_2018_437_MOESM13_ESM.png]

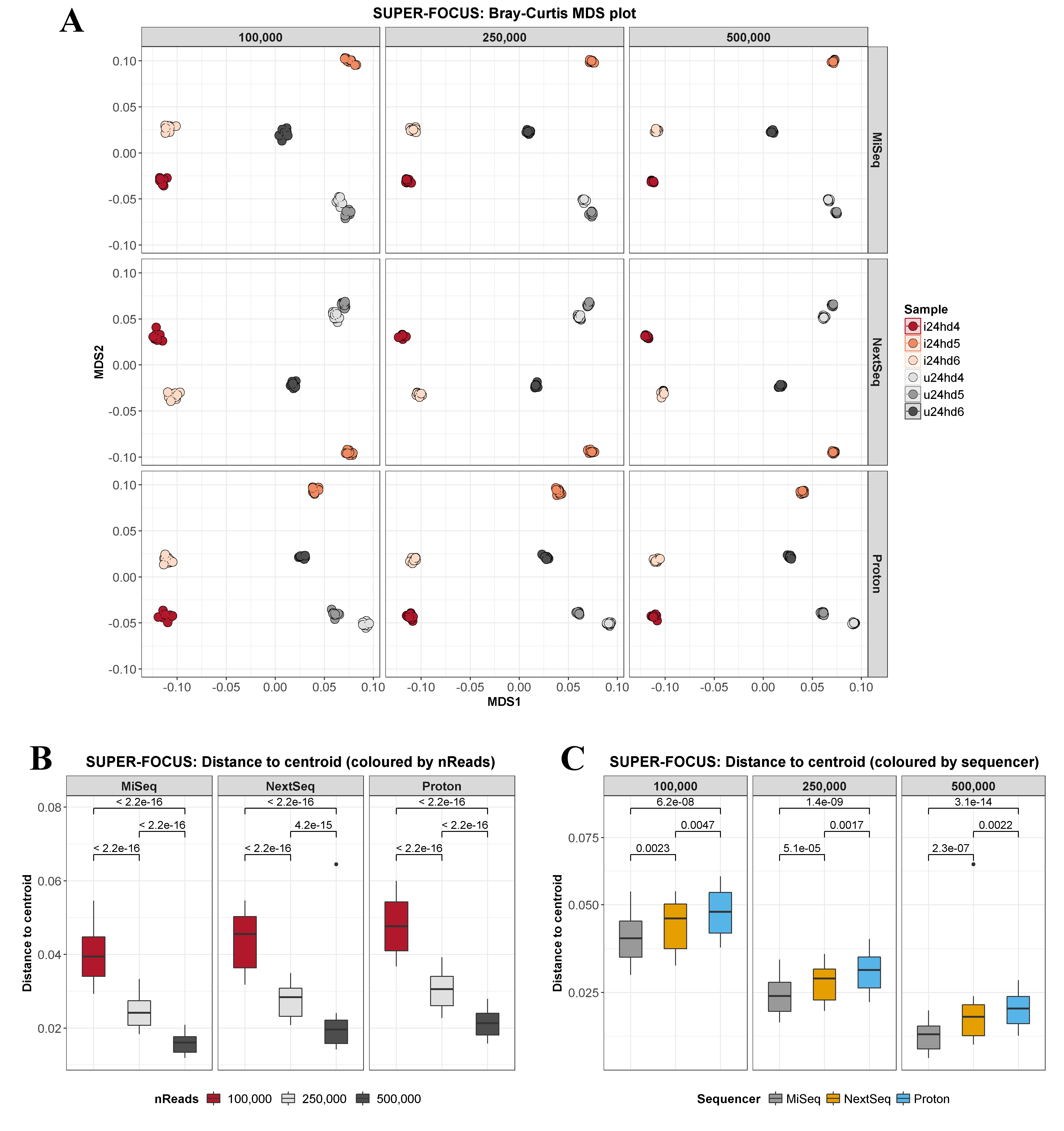

Supplement: Supplementary file 15 — Figure S10. Consistency in the SUPER-FOCUS profiles of randomly subsampled replicates of the same samples. (A) MDS plot (facetted by number of reads) where replicates (coloured by sample) are connected to their respective centroids. (B) The average distance of replicates to their respective centroids at each sequencing depth. (C) The average distance of replicates to their respective centroids for each sequencer. (PNG 202 kb) [file 40168_2018_437_MOESM15_ESM.png]
