# Supplementary material for: Species classifier choice is a key consideration when analysing low-complexity food microbiome data
Source: Microbiome. 2018 Mar 20;6:50. doi: 10.1186/s40168-018-0437-0 (PMC5859664; doi:10.1186/s40168-018-0437-0)
Supplement: Supplementary file 3 — Table S1. Statistical differences in the alpha diversity of kefir samples between the three sequencers. (DOCX 16 kb) [file 40168_2018_437_MOESM3_ESM.docx]

Table S1. Statistical differences in the alpha diversity of kefir samples between the three sequencers.
