# Supplementary material for: Species classifier choice is a key consideration when analysing low-complexity food microbiome data
Source: Microbiome. 2018 Mar 20;6:50. doi: 10.1186/s40168-018-0437-0 (PMC5859664; doi:10.1186/s40168-018-0437-0)
Supplement: Supplementary file 16 — The settings used for each species classifier and PanPhlAn. (DOCX 19 kb) [file 40168_2018_437_MOESM16_ESM.docx]

**SUPPLEMENTAL MATERIAL**

**Compositional analysis**

Here, we outline the commands used for each species classifier, in addition to PanPhlAn, and we describe how these parameters deviated from the default settings. Commands are highlighted in grey.

**CLARK**

ls *.fasta | awk -F '.fasta' '{print "classify_metagenome.sh -O "$0" -R "$0".clark_out -m 0"}' > run_CLARK.sh

sh run_CLARK.sh

ls *.csv | awk -F '.csv' '{print "/shared/software/clarke/1.2.3/estimate_abundance.sh -F "$0" -D $DIR_DB -a 0.1 -c 1 -g 0.05 > "$0".abundances"}' > run_CLARK_Abundances.sh

sh run_CLARK_Abundances.sh

Description: The CLARK classification step was run with full mode execution. The CLARK estimate abundances step was run with minAbundance 0.1, minConfidenceScore 1, and minGamma 0.05.

**Kaiju**

ls *.fa | awk -F '.fa' '{print "/shared/software/kaiju/kaiju/bin/kaiju -t /data/tgsc1/aaron/kaijudb/nr/nodes.dmp -f /data/tgsc1/aaron/kaijudb/nr/kaiju_db.fmi -i "$0" -o "$0"_kaiju.nr.out -z 10 –m 33 -x -v"}' > run_Kaiju.sh

sh run_Kaiju.sh

ls *.nr.out | awk -F '.nr.out' '{print "kaijuReport -u -m 0.1 -t /data/tgsc1/aaron/kaijudb/nr/nodes.dmp -n /data/tgsc1/aaron/kaijudb/nr/names.dmp -r species -i "$0" -o "$0".species.summary"}' > Kaiju_Report.sh

sh Kaiju_Report.sh

Description: The Kaiju classification step was run using the SEG low complexity filter, and the minimum match length was set to 22. Reads were mapped against the RefSeq database. The Kaiju report step was run using minAbundance 0.1. Only classified reads were reported.

**Kraken**

ls *.fasta | awk -F '.fasta' '{print "kraken --threads 10 --preload --db $KRAKEN_DIR/krakken_db "$0" > "$0"_kraken_out"}' > run_Kraken.sh

sh run_Kraken.sh

ls *kraken_out | awk -F 'kraken_out' '{print "kraken-filter --db $KRAKEN_DIR/krakken_db --threshold 0.5 "$0" > "$0".filtered"}' > run_Kraken_Filter.sh

sh run_Kraken_Filter.sh

ls *filtered | awk -F 'filtered' '{print "kraken-mpa-report --db $KRAKEN_DIR/krakken_db "$0" > "$0"_mpa"}' > run_Kraken_Report.sh; sh run_Kraken_Report.sh

Description: Kraken results were filtered using a threshold set to 0.5 to remove low confidence classifications.

**MetaPhlAn2**

MetaPhlAn2 was run using default parameters (<https://bitbucket.org/biobakery/biobakery/wiki/metaphlan2>).

**SLIMM**

ls *trimmed.fastq | awk -F 'trimmed.fastq' '{print "bowtie2 -x $DB_DIR/slimm_db/AB_5K_indexed_ref_genomes_bowtie2/AB_5K -U "$0" | samtools view -bSF4 - > "$1"_mapped_reads.bam"}' > bowtie2_map.sh

sh bowtie2_map.sh

ls *bam | awk -F 'bam' '{print "slimm -m $DB_DIR/slimm_db/slimmDB_5K "$0""}' > run_SLIMM.sh

sh run_SLIMM.sh

Description: Bowtie 2 ([1](#_ENREF_1)) was used to trimmed fastq reads against the slimmDB_5K reference database.

**PanPhlAn**

ls -d *.fasta | awk -F '.fasta' '{print "/shared/software/panphlan/b40c003/panphlan_map.py -c $pangenome_index -i "$0" -o map_results"}' > run_PanPhlAn.sh

sh run_PanPhlAn.sh

panphlan_profile.py -c $pangenome_index -i map_results --add_strains --min_coverage 1 --left_max 1.70 --right_min 0.30 --o_dna result_gene_presence_absence.csv --strain_hit_genes_perc percent_match.txt

Description: The PanPhlAn profiling step was run with a –min_coverage set to 1, --left_max set to 1.7, and –right_min set to 0.3. These parameters increase the tool’s sensitivity.

**REFERENCES**

1. **Langmead B, Salzberg SL.** 2012. Fast gapped-read alignment with Bowtie 2. Nature methods **9:**357-359.
